# Split Membrane: A New Model to Accelerate All-Atom MD Simulation of Phospholipid Bilayers

**DOI:** 10.1021/acs.jcim.4c01664

**Published:** 2025-01-08

**Authors:** Mehrnoosh Khodam Hazrati, Lukáš Sukeník, Robert Vácha

**Affiliations:** † CEITEC—Central European Institute of Technology, Masaryk University, Kamenice 753/5, 625 00 Brno, Czech Republic; ‡ Department of Condensed Matter Physics, Faculty of Science, Masaryk University, Kotlářská 267/2, 611 37 Brno, Czech Republic; § National Centre for Biomolecular Research, Faculty of Science, Masaryk University, Kamenice 5, 625 00 Brno, Czech Republic

## Abstract

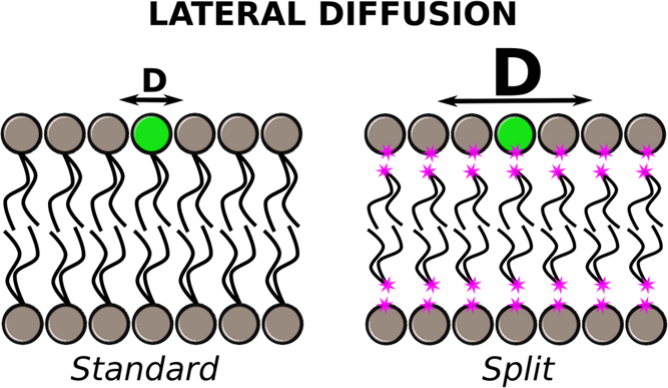

All-atom molecular dynamics simulations are powerful tools for studying cell membranes and their interactions with proteins and other molecules. However, these processes occur on time scales determined by the diffusion rate of phospholipids, which are challenging to achieve in all-atom models. Here, we present a new all-atom model that accelerates lipid diffusion by splitting phospholipid molecules into head and tail groups. The bilayer structure is maintained by using external lateral potentials, which compensate for the lipid split. This split model enhances lateral lipid diffusion more than ten times, allowing faster and cheaper equilibration of large systems with different phospholipid types. The current model has been tested on membranes containing PSM, POPC, POPS, POPE, POPA, and cholesterol. We have also evaluated the interaction of the split model membranes with the Disheveled DEP domain and amphiphilic helix motif of the transcriptional repressor Opi1 as representative of peripheral proteins as well as the dimeric fragment of the epidermal growth factor receptor transmembrane domain and the Human A2A Adenosine of G protein-coupled receptors as representative of transmembrane proteins. The split model can predict the interaction sites of proteins and their preferred phospholipid type. Thus, the model could be used to identify lipid binding sites and equilibrate large membranes at an affordable computational cost.

## Introduction

Cell membranes are predominantly composed of phospholipids, which are amphiphilic molecules self-assembling into a vital semipermeable barrier. There are hundreds of phospholipid species that can differ in headgroups and hydrocarbons, including tail length and degrees of unsaturation.^1,2^ The asymmetric composition of human plasma membranes (PMs) mainly includes sphingomyelin (SM) and phosphatidylcholine (PC) in the outer leaflet, whereas phosphatidylethanolamine (PE) and phosphatidylserine (PS) in the inner leaflet.^3^ However, many other lipids are in low abundance; for example, phosphatidylinositol (PI) and phosphatidic acid (PA) constitute less than 2% of the lipids in PMs.^4^ Such scarce lipids have a crucial role in cell signaling and interaction with specific proteins.^5,6^

With recent developments in computational and molecular force fields (FFs), more realistic modeling of biological cell membranes is now possible.^2,7^ However, to mimic a biological membrane containing a relevant number of low-abundant lipids, a large simulation box is necessary (i.e., 70 × 70 nm^2^ area of membrane).^7−9^ To equilibrate such large membranes, the slow lateral diffusion of lipids becomes the limiting factor. Unfortunately, due to the high viscosity of membranes, lipids have a low lateral diffusion coefficient in the order of (0.5–1.5) × 10^–2^ nm^2^/ns.^10,11^ Therefore, it is not feasible to sample such a large system and adequately capture the dynamics of the interaction between the scarce lipids and proteins on an atomistic level on the microsecond time scale.

To achieve longer time scales, coarse-grained (CG) models are usually employed, and a large membrane composed of 63 different lipids was simulated for 40 μs to mimic the human PM.^8^ Nevertheless, CG models do not capture atomistic details of molecules and do not have the same precision as all-atom (AA) models; hence, simulations with AA models of membrane systems are still necessary.^12−14^

The sampling of heterogeneous biological membranes has been further improved by using the molecular dynamics with alchemical steps (MDAS) method, which allows the study of complex processes, such as ligand binding, protein folding, and chemical reactions.^15,16^ In this method, the alchemical trajectory acts as a Monte Carlo move by changing the positions of randomly selected pairs of lipids.^15^ However, for obtaining meaningful results, this method requires careful parametrization, especially for AA membranes, where sampling of conformational space, selection of appropriate alchemical transformations, and low acceptance probability make MDAS computationally very expensive.^16^

The highly mobile membrane mimetic (HMMM) model is another specialized approach designed to enhance the lateral diffusion of lipids.^17,18^ The model emulates the dynamic properties of biological membranes through the incorporation of lipid-like molecules with a high degree of mobility. The HMMM model shows a very good agreement in the interface region but a low accuracy in the interior of the membrane.^19,20^ Also, users rely heavily on HMMM Builder^21^ due to the complexities involved in building an HMMM simulation system, particularly when dealing with the treatment of lipid tails.

In this paper, we introduce a new “split” AA model of lipids that enhances their lateral diffusion within the bilayer. By splitting lipid molecules into head and tail groups and applying external potentials to maintain the bilayer structure, we accelerate the sampling process. We tested our model for different membrane systems containing various ratios of palmitoylsphingomyelin (PSM), 1-palmitoyl-2-oleoyl-*sn*-glycero-3-phosphocholine (POPC), 1-palmitoyl-2-oleoyl-*sn*-glycero-3-phosphoserine (POPS), 1-palmitoyl-2-oleoyl-*sn*-glycero-3-phosphoethanolamine (POPE), 1-palmitoyl-2-oleoyl-*sn*-glycero-3-phosphatidic acid (POPA), and cholesterol (CHL). We validated our new model by comparing membrane structural properties and lipid–protein interactions with those of the standard AA model. We employed our split model to investigate the interaction of peripheral and transmembrane proteins with membranes. The split model offers the versatility to be easily extended to different lipid types, providing a straightforward approach that accelerates lipid diffusion by a factor of more than ten while keeping the atomistic details. Moreover, the split lipids could be reunited, and the membrane could become whole again.

## Methods

All simulations were carried out using the GROMACS 2021.4 software package.^22−24^ The simulations were performed in the NpT ensemble. The temperature was kept at 310 K using the V-rescale thermostat^25^ with a time constant of 1 ps applied separately for lipids and solvent. In systems with proteins, we used separate thermostats for proteins, lipids, and solvents. Semi-isotropic pressure of 1 bar was applied independently in the membrane plane and along the membrane normal using the Parrinello–Rahman algorithm^26^ with a coupling constant of 10 ps and compressibility of 4.5e-5 bar^–1^. Short-range electrostatics and van der Waals interactions were truncated and shifted to zero at 1.2 nm. Long-range electrostatic interactions were treated with the Particle Mesh Ewald (PME) method,^27^ using a grid spacing of 0.12 nm, real space cutoff of 1.2 nm, and PME order 4. Long-range corrections for energy and pressure were used for dispersion interactions. The geometry of water and all covalent bonds were constrained by SETTLE^28^ and LINCS^29^ algorithms, respectively. The integration time step was 2 fs. Periodic boundary conditions were applied in all directions. After 10 ns equilibration, POPC and POPC/POPS membranes were simulated for 500 ns, while PM mimics were simulated for 1.5 μs.

The studied systems included pure POPC, binary POPC/POPS (1:1 mol/mol), and mimics of the human PM. POPC and POPC/POPS membranes had 64 lipids per leaflet. Mimics contained 600 lipids in each leaflet (total 1200 lipids) composed of PSM, POPC, POPS, POPE, and POPA in different ratios. The lipid ratios are based on the experiments by Lorent et al., and the number of individual phospholipids in the studied systems is listed in Table 1.^30^ As a result, we created three symmetric PM replicas with the same compositions throughout each leaflet. The “inner” and “outer” membranes have the compositions of the inner and outer leaflets of the PM, respectively. The third symmetric membrane is the ”scrambled” membrane, which consists of the phospholipid mixture from the inner and outer leaflets of the PM. In addition, we studied the scrambled membrane containing 50% CHL in both leaflets to investigate the effect of CHL in the split model.

**Table 1:** Number of Individual Phospholipids in the Studied Plasma Membrane (PM) Mimics: Scr Stands for Scrambled Mimics; Scr_chl_ Has the Same Ratios of Lipids in scr but Contains 50% CHL in Both Leaflets

number of phospholipids				
	outer	inner	scr.	scr_chl_
PSM	660	30	348	174
POPC	510	336	420	210
POPS	18	480	252	126
POPE		342	168	84
POPA	12	12	12	6
CHL				600

Systems contained ≈70 water molecules per lipid and Na^+^ and Cl^–^ ions at the physiological concentration of 150 mM with excess ions to neutralize the systems. The initial structures of all membranes were generated by the CHARMM-GUI^31−34^ with CHARMM36 FF^35^ and the TIP3P water model, followed by equilibration at 310 K and 1 bar using default CHARMM-GUI parameters. However, this step is not typically required for preparing the initial structure inputs for the split model. Our split model is based on a modified Slipids FF.^13,36,37^ Therefore, we simulated the membranes with our split Slipids FF as well as standard Slipids FF.

**Figure 1:**
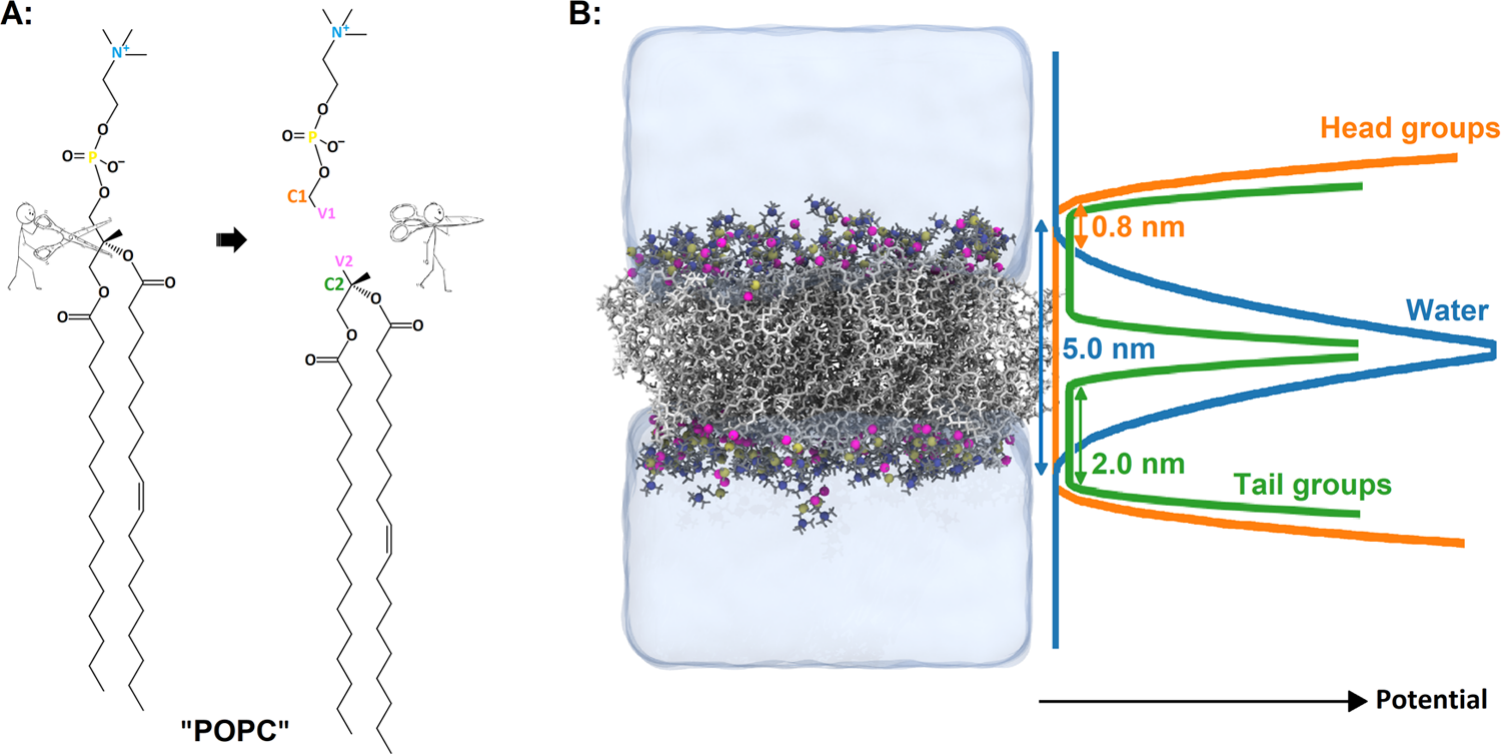
(A) Illustration of the POPC lipid and its split model: head and tail groups are separated into two moieties at glycerol. The split carbon bond is ended with a virtual site (V1 for headgroups and V2 for tails); (B) snapshot of the POPC/POPS membrane after 500 ns simulation using the split model. For clarity, lipid phosphorus is highlighted in yellow, nitrogen atoms in blue, and virtual sites in pink, while water and lipid tails are colored light blue and gray, respectively. To keep the bilayer intact, potentials are applied: (1) flat-bottomed potentials on C1 and C2 atoms of glycerol moieties (orange and green curves, respectively) and (2) an inverted flat-bottomed potential on oxygen atoms of water molecules (blue curve).

**Figure 2:**
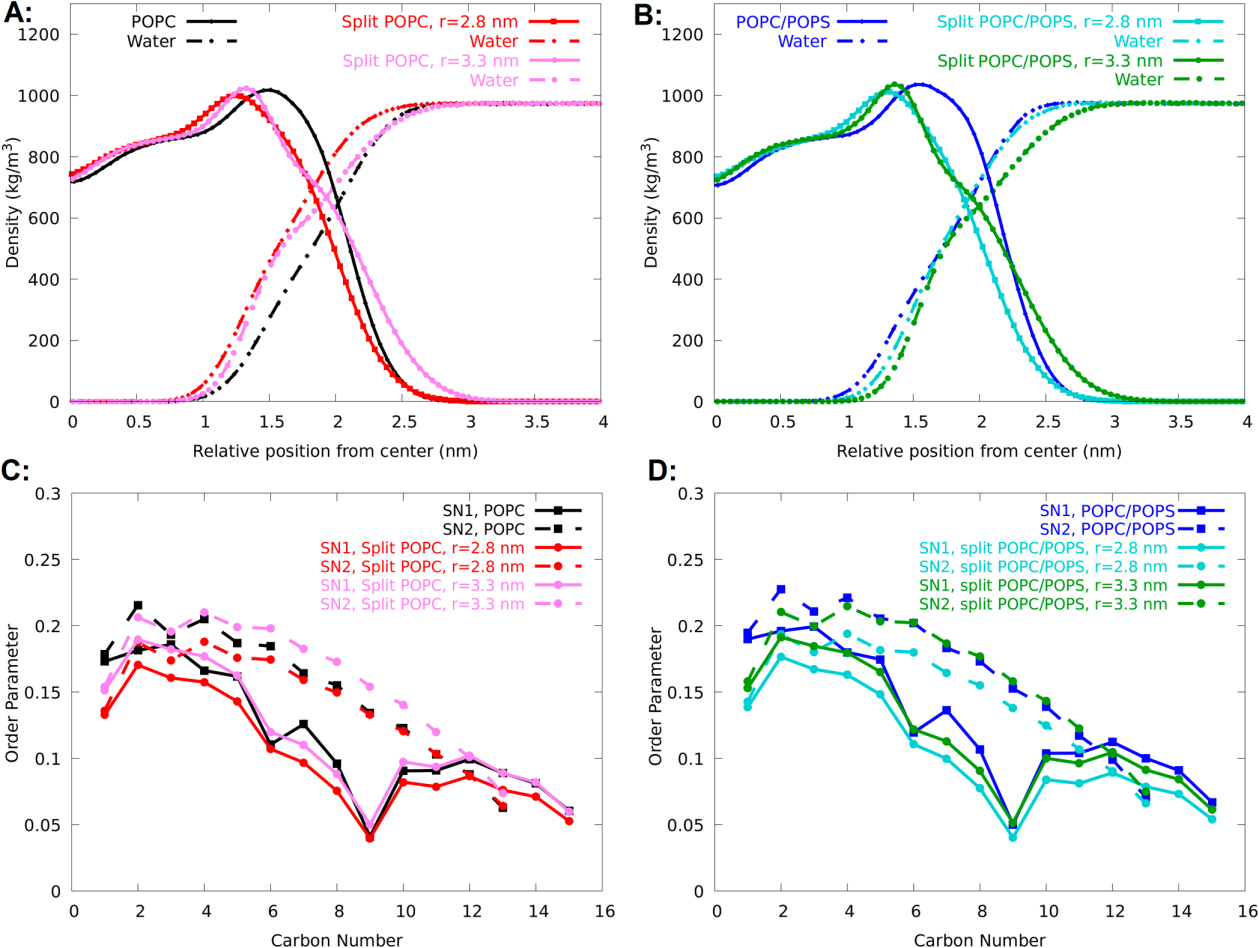
Membrane structural properties comparison for the standard Slipids FF and the split Slipids FF. Density profiles for (A) pure POPC and (B) binary POPC/POPS. Tail order parameters (SN1 and SN2) for (C) pure POPC and (D) binary POPC/POPS. The restraint with a distance of 2.8 nm reproduced better density profiles, while 3.3 nm yielded better tail order parameters. Overall, the displayed profiles show minor differences that did not impact lipid density profiles and acyl tail order parameters between membranes simulated with standard Slipids and split Slipids FF.

Each lipid is split into the head and tail moieties at the glycerol carbon bond, leading to phosphate (see Figure 1A). Virtual sites with no mass and no electrostatics or Lennard–Jones (LJ) interactions were applied to the cut bond to keep its valence. To preserve the bilayer structure, a set of flat-bottomed positional restraint potentials was applied (see Figure 1B). The oxygen atoms of water were restrained using an inverted flat-bottomed potential with a force constant of 2.5 kJ/mol at a distance of 2.5 nm from the center of mass (COM) of the membrane. Additional flat-bottomed potentials were applied to the last atoms of the head (C1) and first atoms of the tail (C2) groups where the lipid was cut. The force constant and the distance from the COM of the membrane were 10 kJ/mol and 3.3 nm for C1, and 50 kJ/mol and 1.0 nm for C2, respectively. Only for the pure POPC, we used a tighter restriction distance of 2.8 nm on the headgroups. These parameters were selected to reproduce density profiles, area per lipid, acyl tail order parameters, and P–N angle of all-atom membrane simulated by standard Slipids FF (Figures 2, S1, and S2). We calculated the area per lipid by dividing the area of the simulation box in *xy*-plane by the number of lipids in each leaflet. We used the GROMACS tool *gmx order* to calculate the acyl tail order parameters. The lateral diffusion coefficients were determined by using eq 1.^38^
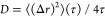
1where τ is the simulation time range during which the lateral mean squared displacement (MSD), (Δ*r*)^2^, is calculated.

We employed the split model to investigate the interaction of membranes with proteins. As a representative of peripheral proteins sensitive to lipid composition, we selected the DEP domain from the human Disheveled protein and the amphiphilic helix motif of the transcriptional repressor Opi1. The DEP domain was shown to adhere to membranes containing POPS lipids but not pure POPC membranes.^39^ We prepared the initial DEP domain structure as in the original study.^39^ The structure of the DEP domain from human DVL3 was modeled using the crystal structure of the DEP domain from mouse Dvl2 (PDB ID: 3ML6(40)), which shares a high sequence identity (90%). Necessary mutations were manually introduced with PyMOL.^41^ A single replica of the domain was then positioned approximately 1.5 nm from each leaflet of the equilibrated lipid bilayers. Opi1 was shown to have a preference for negatively charged lipids (i.e., POPA or POPS). The Opi1 peptide was generated by AmberTools18.^42^ To ensure symmetry in the systems and evaluate the convergence, we placed an identical image of the DEP domain/Opi1 on the lower leaflet. We employed the Amber99SB-ILDN FF for the DEP domain and Opi1.^43^ For this study, we selected a binary POPC/POPS (1:1 mol/mol) membrane with 64 lipids per leaflet.

To study transmembrane proteins, we decided on the dimeric fragment of the epidermal growth factor receptor transmembrane (EGFRtm) domain. The EGFRtm domain comprises 44 residues (from E^634^ to R^677^). The initial structure of the EGFRtm dimer was obtained from the Protein Data Bank Web site ((PDB: 5LV6)). A binary POPC/POPS (1:1 mol/mol) membrane with 180 lipids per leaflet was selected for this study. We used CHARMM-GUI to prepare the initial configuration of the protein and the membranes.^31−34^ The systems were equilibrated for 10 ns, followed by 1.5 μs MD production runs.

To further check the reliability of the split model for transmembrane proteins, we selected the human A2A adenosine of heterotrimeric guanine nucleotide-binding protein (G protein-coupled receptors, GPCRs) (PDB ID: 3EML) because GPCRs are the largest family of membrane proteins.^44^ The receptor was embedded in a POPC/POPS (7:3 mol/mol) to match the experimental ratio.^45^ The bilayer was constructed with 180 lipids (126 POPC and 54 POPS) per leaflet using the CHARMM-GUI membrane builder. The systems were equilibrated for 10 ns, followed by 1.5 μs MD production runs, as in the case of the EGFRtm domain. We carried out MD simulations with the standard and split models.

Finally, we prepared a Python code (*Merger.py*) to reunite the head and tail groups and make the membrane whole again. When reuniting the head and tail groups, we select the closest head and merge them. Sometimes, the reunification connects headgroups and tails that are not very close to each other, which causes the system to become significantly out of equilibrium and needs to be minimized or shortly re-equilibrated. In such a re-equilibration, we fixed the positions of the headgroups and forced the acyl tails to move toward their heads. Following the reattachment of the head and tails, it is recommended to minimize and re-equilibrate the membrane with *xy*-restrains on headgroups. We reunited the head and tail groups in the final configurations of the split membranes and re-equilibrated the systems for 500 ns. For the EGFRtm domain system, we extended the equilibration time to 1.5 μs.

## Results

### POPC and POPC/POPS Membranes

**Figure 3:**
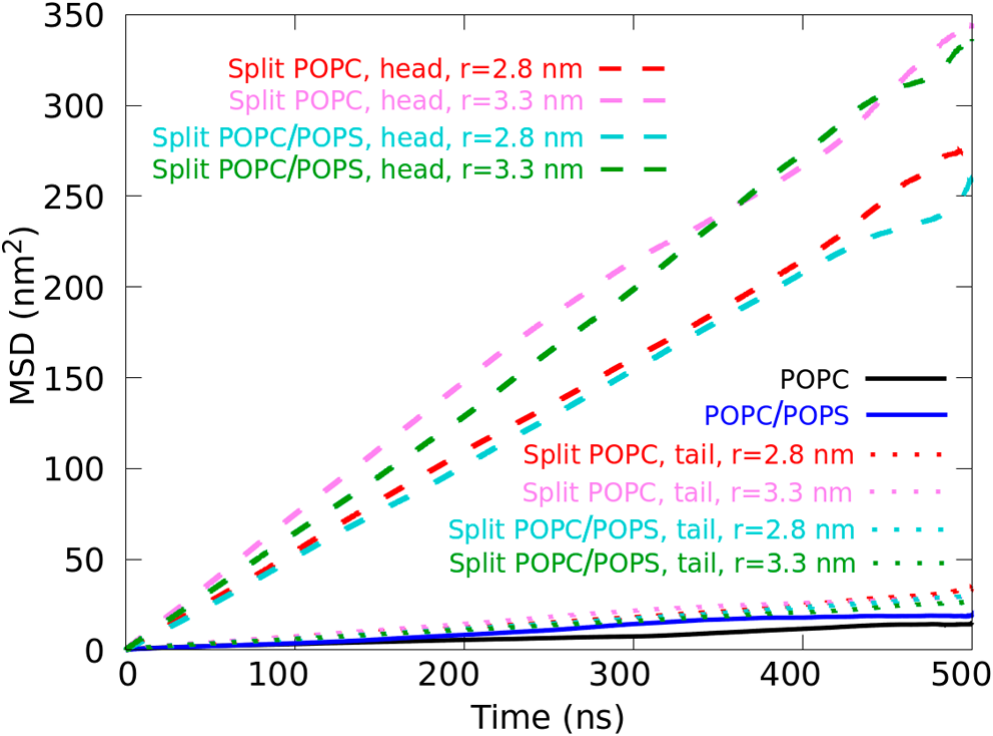
Speedup of lipid diffusion for the split model demonstrated by mean square displacement (MSD) analysis of full lipids (solid line), split headgroups (dashed line), and split tails (dotted lines). Split POPC and POPC/POPS systems are compared to POPC and POPC/POPS simulated by the standard Slipids all-atom FF. The speedup is more than ten times for the split headgroups.

First, we tested numerous different restraints to find the optimal values of restraints for POPC and POPC/POPS (1:1 mol/mol) bilayers; see Figure S2 for an example. We found a water restraint with a force constant of 2.5 kJ/mol and a distance of 2.5 nm from the membrane COM yielded the most accurate density profiles for water. We applied a restraining distance for tail groups (on C2 atoms) in all split systems to avoid the flip-flopping of the tails. As can be seen from Figure 2A,B, for headgroups, using a restraining distance of 2.8 nm and a force constant of 10 kJ/mol resulted in more accurate density profiles. However, for the binary POPC/POPS system and all other mixed systems, we decided to use a looser restraint distance of 3.3 nm, which also better reproduced the tail order and area per lipid. In addition, this more distant restraint also increased the diffusion coefficient by about 1.5 times compared to the more restricted (2.8 nm) model (see Figures 2B,D, 3, and S1B,D).

For the split membranes, the acyl tails are slightly more disordered near the glycerol region, where the lipids are split. The tail disorders in the split membrane models are almost identical to the standard models in the bilayer center (below the fifth carbon). Interestingly, there is a tail order difference above the double bond in the SN1 chain (around the seventh carbon). The split models showed a negligible difference in the average area per lipid compared with that of standard models. The average area per lipid for split POPC and split POPC/POPS are 0.658 and 0.625, respectively, and are within error from the standard membranes; see Figure S1A,B. The area per lipid was calculated by dividing the membrane’s total area by the number of lipids, thus reflecting mainly the correct area of the simulated membrane patch. The average P–N angles for split and standard POPC membranes were almost the same (90.17 and 90.32°). Similarly, for POPC/POPS, the average P–N angles were 89.81 and 92.03° for the split and standard models. The distributions are approximately normal; see Figure S1C,D. Figure S1E,F shows the membrane thickness using P and N atoms of the headgroups and C2 atoms of the tails as references for the standard and split models of pure POPC and binary POPC/POPS, respectively. No matter which atom we consider for the calculations, the membrane thickness in the split model is very similar to that in the Standard Model. Overall, there are minor differences in average density profiles, acyl tail order parameter, area per lipid, P–N angle distribution, and membrane thickness between membranes simulated with standard Slipid and split Slipid models of POPC and POPC/POPS membranes. Therefore, the split model can reproduce the structural features of the membrane.

Using eq 1, we calculated the lateral diffusion coefficients from 50 to 400 ns of the simulation time during which the MSD curve is linear. Our split model increased the lateral diffusion coefficients for the headgroups by more than 10 times (see Figure 3 and Table 2). In the split model, the headgroups do not remain attached to their respective tails, and they act as free molecules. This is the main reason for the acceleration of diffusion. As expected, the acyl tails also diffuse slightly faster than the whole lipids. However, because the acyl tails are relatively large hydrophobic molecules, they still diffuse quite slowly, and the increase of diffusion is relatively small. In the studied membrane, the lipid tails were the same for all lipids. Therefore, enhanced diffusion of tails was not our concern and the aim of the model.

**Table 2:** Diffusion Coefficients of POPC Lipids in POPC and POPC/POPS Membranes (nm^2^/ns) to Demonstrate the Enhanced Diffusion of the Split Model, Where the Diffusion of Head and Tail Groups Were Evaluated Separately, in Comparison to the Standard Slipids All-Atom FF

system	*D* × 10^^2^^ (nm^2^/ns)	time^1^ (μs)	speedup
POPC	0.75(±0.05)	13.33	
split head	8.84(±0.37)	1.13	11.8
split tail	0.93(±0.13)	10.75	
POPC/POPS	0.80(±0.14)	12.50	
split head	9.20(±1.22)	1.09	11.5
split tail	0.83(±0.02)	12.05	

1 The simulation time needed for a single lipid molecule to diffuse on average 20 nm^2^.

### DEP Domain at POPC and POPC/POPS

To investigate the applicability and efficiency of our model, we used it to study lipid–protein interactions. As a representative of peripheral proteins, we selected the Disheveled DEP domain, which consists of 87 amino acids and has been shown to have a preference for negatively charged lipids, such as POPS or POPA.^39^

**Figure 4:**
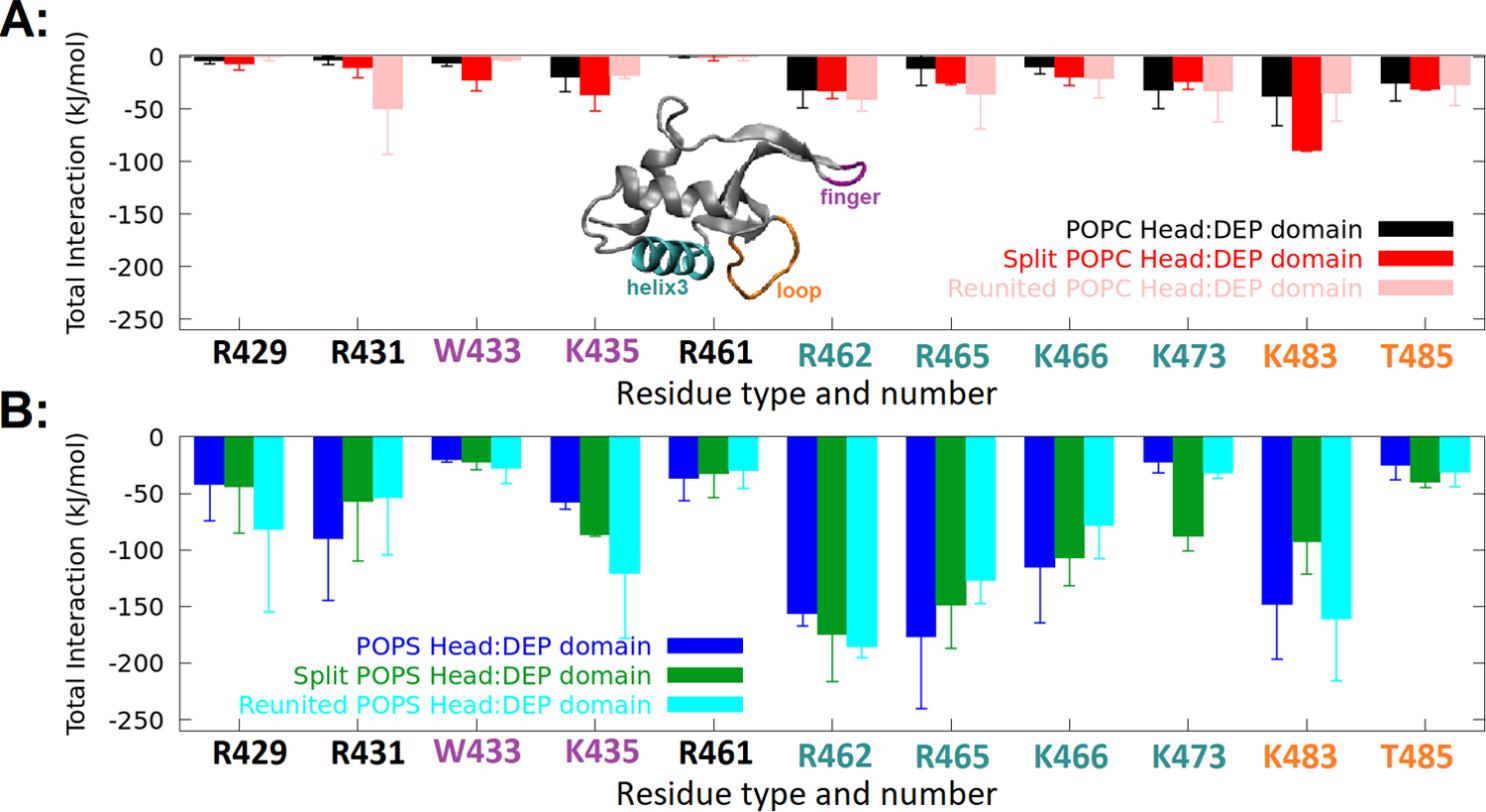
Comparison of average total (LJ + Coulombic) short-range interactions of the most interacting residues of DEP domain with (A) POPC headgroups and (B) POPS headgroups in the binary POPC/POPS systems. The values obtained from the simulation by standard, split, and reunited FFs are mostly within errors, which suggests that similar interaction sites were predicted by all three models.

In Figure 4, we compared the average of total (Coulombic + LJ) short-range interactions between the most interacting residues of the DEP domain and binary POPC/POPS membranes using standard, split, and reunited models. We found that three distinct DEP domain regions (helix3, finger, and loop) are predominantly involved in membrane binding. These regions contain positively charged amino acids, arginine (R) and lysine (K), which primarily interact with the negatively charged POPS headgroups. Arginine and lysine residues in helix3 (R462, R465, K466, and K473), lysine residue in the loop (K483), the polar residue with an aromatic substituent (W433) of the finger, and also two basic residues (R429 and R431) comprise the binding sites of DEP to the membranes. Figure S3A shows the average of total short-range interactions between the most interacting residues of the DEP domain and pure POPC membranes. In conclusion, the split model could predict the interaction sites of the DEP domain in good agreement with the standard FF and previous reports.^39^

The LJ and Coulombic interaction potentials of the most interacting residues for the pure POPC and binary POPC/POPS systems are shown in Figures S3B,C and S4, respectively. Tables S1–S4 summarize the strength of the interactions between the DEP domain (averaged over both copies in the system) and phospholipids headgroups. The tables also include the sum of short-range Coulombic and LJ interaction energies between DEP and the studied membranes. In the binary standard and split systems, the interaction of DEP domains is much stronger with POPS headgroups compared to that with POPC headgroups, demonstrating the domain preference for POPS. The summed interactions between DEP domains and phospholipids were stronger in the split models compared to the standard Slipids FF. The origin of the difference in the interaction strength is discussed below.

To further investigate the stronger interactions of POPC headgroups, we compared the insertion depth of DEP domains into studied membranes by measuring distances between the COM of helix 3 of the DEP domains and the COM of the membranes. Figure S5A,B shows the fluctuations of helix3 COM compared to membrane COM during 500 ns of simulation for pure POPC and the binary POPC/POPS systems, respectively. Table 3 summarizes the results for the studied systems in the last 50 ns when the DEP domain was adsorbed on all membranes. The results show that in both pure POPC and binary POPC/POPS, the insertion depths of the helix3 COM in the reunited systems are within the error between the standard and split models. The average insertion depths differ only within error between the POPC/POPS and the split POPC/POPS bilayers. Note that the differences are higher for POPC and split POPC systems because one of the domains was desorbed from the membrane for about 200 ns during the simulation of the standard system. We observed a similar desorption also for the reunited membrane. Despite the interactions being stronger in the split model, the depth of the DEP domain insertion is comparable between the models.

**Table 3:** Average Insertion Depth for the Helix3 COM of the DEP Domains in POPC and POPC/POPS Membranes in the Last 50 ns of MD Simulation^a^

Helix3 COM distances from membrane COM (nm)						
	POPC	split POPC	reunited POPC	POPC/POPS	split POPC/POPS	reunited POPC/POPS
DEP 1	3.28	2.83	3.09	2.92	3.05	3.05
DEP 2	3.07	2.90	2.96	3.07	2.54	2.74
average	3.18	2.87	3.03	2.99	2.79	2.90
diff.	±0.11	±0.04	±0.07	±0.07	±0.25	±0.16

a Diff are the difference between the values for the two DEP domain images. The difference in the insertion depths for all models is similar. It is about 0.3 nm or less between the split and standard models, indicating a slightly deeper insertion of domains in the split model. The domains in the reunited model also had deeper insertion but only by 0.1 nm compared with the standard model.

Figure S6 displays the radial distribution function (RDF) between the DEP domain surface and P atoms of POPC and POPS in binary POPC/POPS systems. RDF profiles confirm the preference of the DEP domain for negatively charged POPS over POPC lipids and show a stronger interaction with the POPS phosphate groups in the split POPC/POPS compared to the standard membranes. The reunited system exhibited a domain-POPS interaction strength that was intermediate between the split and standard models. However, the interaction between the POPC phosphate groups and the DEP domain was stronger in the split model than in the standard and reunited models due to the desorption of one of the domains in the standard and reunited models during the simulation time (Figure S5A).

Lipid–protein interactions are more numerous and stronger in the split model because the headgroups are free to reorient. When the membrane is reunited, it is expected that the interaction energies between the protein and headgroups will drop. Indeed, the interaction is very similar in the reunited and standard models for DEP on the POPC/POPS membrane. In contrast, the values are significantly different at the POPC membrane, which is likely due to low sampling of the different metastable states for these systems, which, apart from diffusion, include slow domain reorientation, see Figure S7.

In summary, the split model captured the main features of the interaction between the DEP domain and phospholipids. These features include the domain preference for anionic POPS lipids and the same membrane binding sites of the DEP domain as obtained with the standard Slipids FF.^39^ Compared to the standard model, the split model produces configurations with stronger interactions between the domain and headgroups. The stronger interactions with POPC headgroups persisted even after the split membrane was reunited and simulated for 500 ns in different replicas. Therefore, further investigation is required to determine the equilibrium configuration. Nevertheless, the split model can be used to generate new initial conformations and, in particular, to predict the protein preference for a specific phospholipid in mixed membranes (approximately 10-fold faster).

### Opi1 Amphiphilic Helix at POPC/POPS

**Figure 5:**
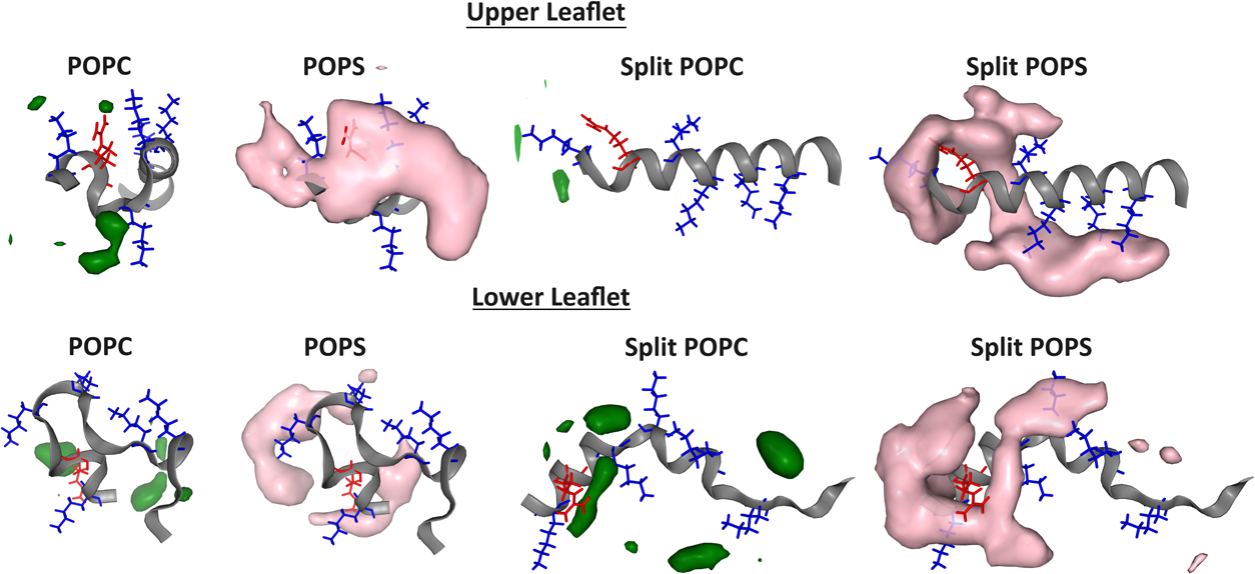
Time-averaged density of P atoms from POPC and POPS lipids in the upper and lower leaflets around the amphiphilic helix of Opi1 in standard and split models are presented. The green and pink volumes indicate the local density of P atoms of POPC and POPS lipids throughout the trajectory, respectively. The last snapshot of the amphiphilic helix of Opi1 is shown to highlight the hotspots of lipid binding. The arginine residue is depicted in red, while the lysine residues are shown in blue. POPS shows higher density around the arginine residue, particularly in the split model.

With previously identified selectivity of negatively charged lipids,^46^ we proceeded to validate our split model using the amphiphilic helix of Opi1 at the POPC/POPS (1:1 mol/mol) membrane. The basic residues in the Opi1, i.e., arginine and lysine residues, were found to form salt bridges with the lipid headgroups.^47^Figure 5 shows the density volumes of P atoms of POPC and POPS around the Opi1 amphiphilic helix for the standard and split models calculated throughout 1.5 μs of simulation. There are higher densities of POPS lipids around the helix. We confirmed the selectivity by calculating the RDF distributions. Figure S8 shows RDFs of lipid P atoms around the helix using standard, split, and reunited models. The previously observed selectivity for POPS over POPC lipids is captured in all models. The strongest helix binding to POPS is in the split model, followed by the reunited and standard models. Considering the upper and lower leaflets as two separate replicas, the split and reunited models show a result that is more consistent than that of the standard model.

### EGFRtm Dimer in POPC/POPS

**Figure 6:**
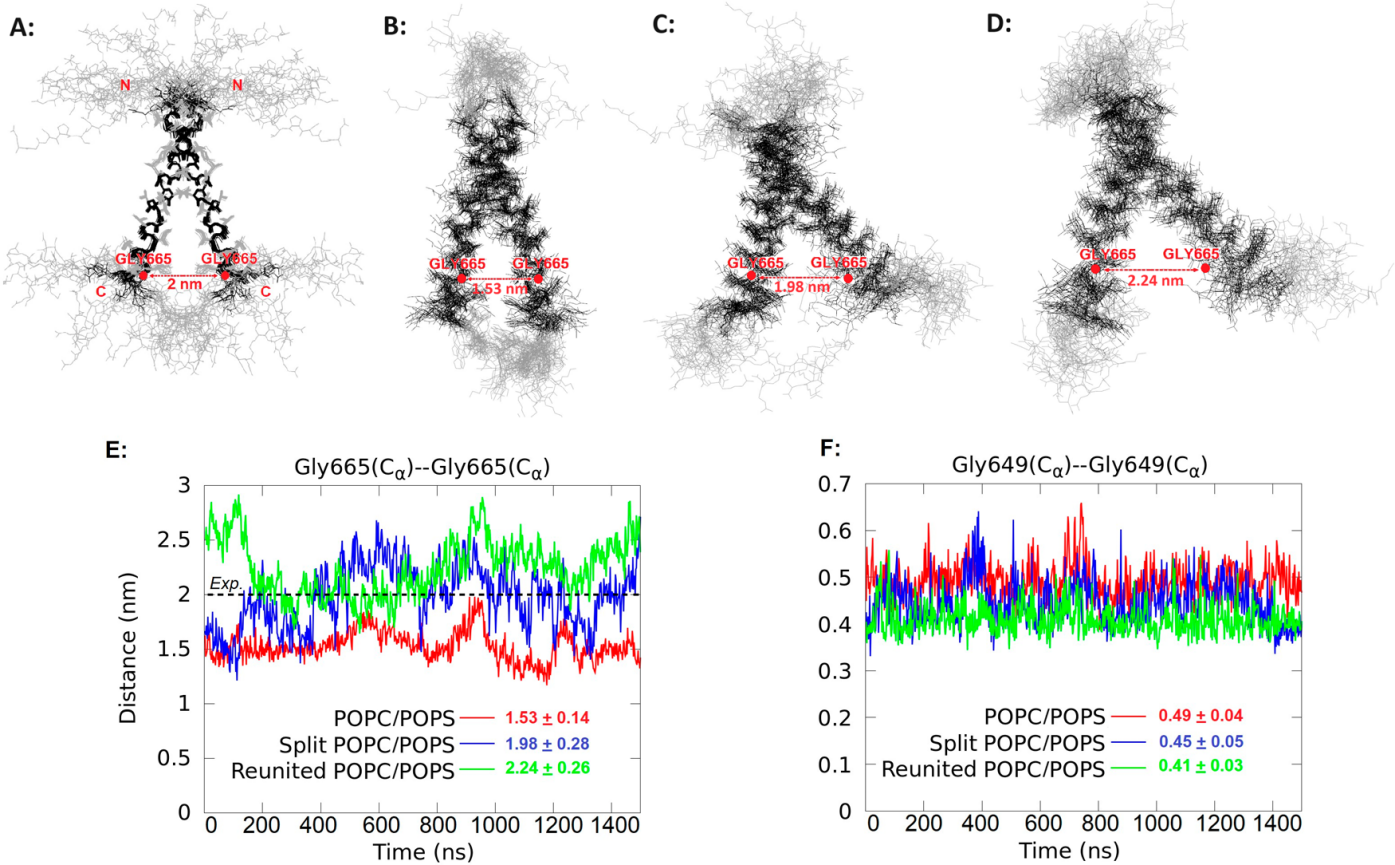
Comparison of EGFRtm structures obtained from the MD simulation results and experiments. (A) Estimated 20 NMR structures of the EGFRtm dimer in bicelles are shown by heavy atoms of TM domains (K^642^–R^671^)_2_ in black and flexible JM regions (E^634^–P^641^ and H^672^–R^677^)_2_ in gray. (Image is adapted with permission from Bocharov et al.^48^ Copyright 2017 American Chemical Society.) 20 representative random snapshots of the EGFRtm dimer during the simulation time in the (B) standard POPC/POPS, (C) split POPC/POPS, and (D) reunited POPC/POPS. To facilitate comparison, the C_α_ of the dimers are aligned, and the approximate positions of C_α_ of GLY665 residues of the last snapshot are highlighted by red spheres. (E, F) Distances between the C_α_ of GLY665 and GLY649 residues during the 1.5 μs of simulations of the EGFRtm N-terminal dimer conformations embedded into the POPC/POPS bilayer in standard, split, and reunited membranes. For the GLY649-GLY649 C_α_ distances, all models show oscillations between 0.4 and 0.6 nm, whereas, in experiments, fluctuations around 0.5 nm are reported. The split model predicted the 2 nm distance for GLY665-GLY665 residues, in good agreement with the experiment. Consequently, the split model demonstrates an improved equilibrium.

To investigate the applicability of our model to study the transmembrane proteins, we selected the EGFRtm dimer as a representative for transmembrane proteins and studied the dimer in the POPC/POPS (1:1 mol/mol) membrane using the standard and split models. The selection was motivated by the small size of the protein and the available experimental data. Figure 6A shows the representative ensemble of EGFRtm dimer structures obtained by nuclear magnetic resonance (NMR).^48^ These structures in the N-terminal transmembrane (TM) state, i.e., with N termini together, showed a distinct hydrophobic helical structure with highly flexible N- and C-terminal juxtamembrane (JM) regions in bicelles.^48^

Figure 6A displays the initial conformation. Figure 6B,C shows the representative conformations from the end of 1.5 μs of simulations using the standard and split models. In addition, we further conducted a 1.5 μs simulation of the reunited membrane, starting from the last configuration of the EGFRtm dimer in the split model simulation, see Figure 6D.

Throughout all simulations, the N-terminal state of the dimer prevails, which is in line with previous findings (Bocharov, 2017;^48^ Arkhipov, 2013^49^). The structure obtained from the end of the reunited membrane is similar to the structure obtained from the split model. Moreover, both of these structures are closer to the experimental structure than the one obtained from the simulations with the standard model, suggesting that the split model can be used to accelerate the equilibration of transmembrane proteins.

Figure 6E,F shows the fluctuations of intermonomeric distance between C_α_ of GLY665-GLY665 and GLY649-GLY649 residues. In the split model, the distance for GLY665-GLY665 residues is about 2 nm, in good agreement with the experiment.^48,49^ In contrast, in the standard model, the dimer has a distance of 1.5 nm between the GLY665-GLY665 residues. In both split and standard models, the GLY649-GLY649 C_α_ distances oscillate between 0.4 and 0.6 nm, in line with the experiment.^48,49^ The results from the reunited system are more consistent with those of the split model. Our results suggest the equilibrium state might not be attainable on the time scale of a few microseconds of simulation using the standard membrane, and our split model could enhance the equilibration.

**Figure 7:**
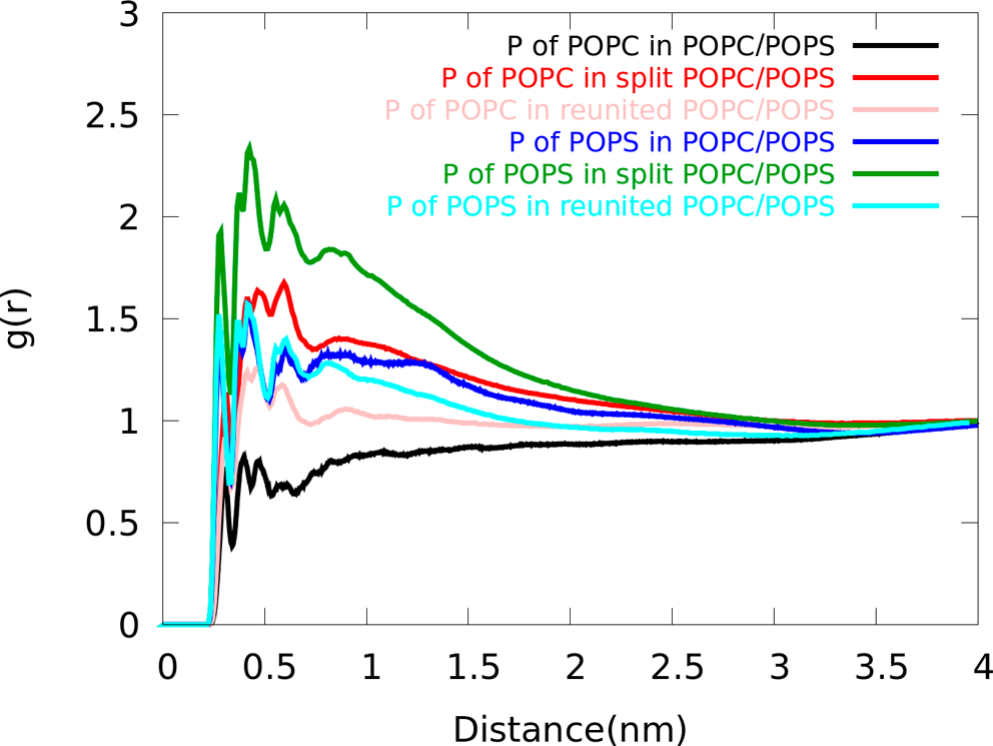
Radial distribution functions for P atoms of headgroups around the EGFRtm dimer in the POPC/POPS membrane using standard, split, and reunited models during 1.5 μs of MD simulation. In all three models, the interaction of POPS headgroups with dimer is stronger compared to POPC headgroups.

Finally, we compared the interactions between the dimer and lipid headgroups. Figure 7 shows the RDF of headgroup phosphate around the EGFRtm dimer during 1.5 μs of MD run for standard, split, and reunited POPC/POPS models. In all systems, POPS headgroups interacted more strongly with the dimer, indicating the tendency of positively charged arginine and lysine residues of the dimer to interact with negatively charged POPS headgroups. Similar to the DEP domain, the interactions between the protein and phospholipid headgroups are stronger in the split membrane. However, after reuniting the membrane, the interaction with POPS lipids decreased to the level found in the standard membrane, while the interaction with POPC headgroups remained stronger. Nevertheless, the dimer structures obtained in both split and reunited models exhibited a closer resemblance to experimental counterparts than the structure derived from the standard model.

### GPCR in POPC/POPS

**Figure 8:**
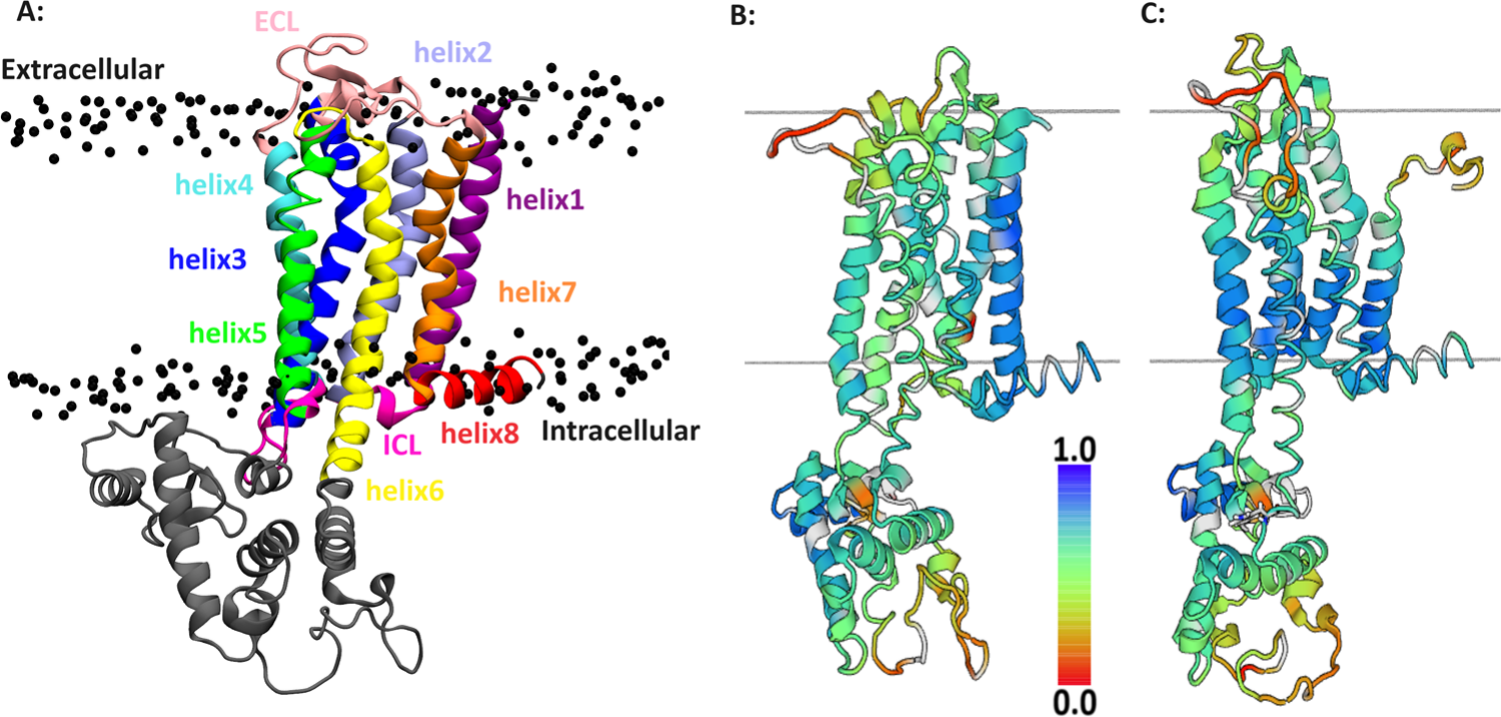
Structures of the A2A adenosine receptor. (A) Crystal structure with each helix, ICL, and ECL are differently colored. P atoms of the membrane are colored in black. (B, C) Receptor structure in the POPC/POPS membrane after 1.5 μs simulation is colored according to the local distance difference test (LDDT) versus the crystal structure as a reference. Blue represents the highest LDDT score, whereas red represents the lowest score. The approximate positions of the upper and lower leaflets of the membranes are shown as gray lines. B is for the simulations with the standard force field, while C depicts results using the split lipid model.

The human A2A adenosine receptor belongs to the class of heterotrimeric GPCRs and is a transmembrane protein of 448 amino acid residues that facilitates the crucial function of extracellular adenosine in numerous physiological processes.^46^ GPCRs are the largest family of membrane proteins and represent a promising route for the development of therapeutic targets for a wide range of diseases.^44^ We have investigated a POPC/POPS (7:3 mol/mol) system to assess the ability of the split model to differentiate the GPCR structure in a bilayer. Figure 8A shows the crystal structure of the human A2A adenosine receptor. The transmembrane domain consists of eight α helices, which are helix1(Gly5–Trp32), helix2(Thr41–Ser67), helix3(His75–Arg107), helix4(Thr119–Leu140), helix5(Asn175–Ala204), helix6(Arg222–Phe258), helix7(Leu269–Arg291), and helix8(Arg296–Leu308). The intracellular loops (ICLs) are Leu33–Val40, Ile108–Gly118, and Leu208–Ala221, while the extracellular loops (ECLs) are Thr68–Cys74, Leu141–Met174, and Cys259–Trp268. Figure 8B,C shows a local distance difference test (LDDT) of the Cα atoms of the adenosine receptor relative to the crystal structure by 1.5 μs of simulation in the standard and split POPC/POPS, respectively. In the standard model, the greatest deviation from the crystal structure is observed in helix 2. In the split model, the largest deviation from the crystal structure is observed in helices 1. Both models show deviation from the crystal structure in the extracellular loops (ECLs). Overall, the LDDT scores for the split model are in good agreement with the standard model. Figure S9 shows the RMSD during 1.5 μs of simulation in the standard and split POPC/POPS. Both models exhibit an RMSD of approximately 2.95 nm. In conclusion, the split model was able to predict the structure of the protein with a margin of error compared with the crystal structure. Figure S10 shows the RDF profiles for P atoms of POPC and POPS in the upper and lower leaflets for the standard, split, and reunited models. There is good agreement among all models.

### Plasma Membrane

To investigate the ability of our split model to be employed for systems containing different lipid types, we used the split model to simulate mimics of human plasma membranes (PMs). The PMs include symmetric mimics made of an outer leaflet, an inner leaflet, and a scrambled variant. The lipid compositions are based on the experiments by Lorent et al.^30^ (for details, see Table 1).

Figure S11A–C compares the density profiles for the split and standard Slipids FF. Figure S12A–F shows the RDF of lipid head and tail moieties with respect to water. Except for the PE headgroups aggregation in the inner leaflet mimic, our results from the split model are in good agreement with the standard Slipids FF. The PE headgroups are less space-filling, which could lead to artifacts.^50,51^ In addition, PE headgroups tend to form h-bonds between the negatively charged phosphate and the positively charged “naked” NH_3_^+^ group. These interactions become an issue when the membrane contains a high ratio (more than 20%) of PE lipids. To overcome the PE aggregation, one can probably use the systematic refinement of nonbonded fix (NBFIX) parameters previously introduced for CHARMM and AMBER FFs by Yoo et al.^52,53^ However, fixing the split Slipids FF for PE (and possibly also PA) headgroups is out of the scope of the current work. Therefore, we recommend our current split model for membranes with a low content of POPE or/and POPA (not more than 15%).

To evaluate the effect of CHL in our split model, we studied a scrambled PM containing 50% CHL in both leaflets (*scr in Table 1). Density profiles and RDFs are shown in Figure S13. The results demonstrate that the split model is also suitable for use in systems containing CHL.

The split FF accelerated diffusion more than 20 times on average. The diffusion coefficients are about 8 nm^2^/ns and vary slightly, depending on the lipid ratios in the system. The diffusion coefficients for PM models are summarized in Table S5. In conclusion, the split model is suitable for the simulation of PM mimics and systems containing a CHL.

## Discussion

With the increasing complexity and size of systems under investigation in computer simulations, it is important to properly equilibrate and sample the systems. However, the slow lateral diffusion of lipids makes such simulations with membranes computationally expensive. To address this problem, we have developed a split model that accelerates lateral diffusion in bilayers more than 10-fold while capturing the atomistic details of lipids.

The model is based on splitting lipid molecules into two smaller moieties, a hydrophilic headgroup and hydrophobic tails, which are kept in the bilayer by restraining potentials. These restraints likely inhibit large-scale undulations of membranes but are important to preserve their structural stability. We carefully calibrated the parameters of restraints to match the membrane density profiles as well as the area per lipid. While the model significantly enhances the diffusion of headgroups, the diffusion of lipid tails remains relatively unchanged. In the model, we restrained the water molecules to maintain the correct density profiles of the pure lipid membrane. This restraint will affect the water permeation through the membrane and also may affect water interaction with deeply inserted protein residues. The split model thus could be suitable for the study of transmembrane proteins with a hydrophobic core, which is supported by our study of the EGFRtm dimer. Note that due to the higher mobility of lipid headgroups, they can also reorient more easily than in the standard parametrization. Therefore, the atypical distribution of headgroups around proteins should be investigated using the equilibrated reunited membrane.

A similar approach has been adopted by Tajkhorshid et al., who developed the HMMM model.^17^ The HMMM model mimics the properties of biological membranes by using shorter lipids and lipid-like solvents in the lipid tail region. The split model may seem similar to the HMMM model, but the principles and methodology are completely different. Our model of a membrane offers a more realistic representation of the internal aspects of membranes (acyl tails) compared to the HMMM model, where the inner part of the membrane is filled with an organic solvent. The lipid lateral diffusion for the split model is in the similar range as for HMMM model membranes, being (8.4–12.6) × 10^–2^ nm^2^/ns.^18^ Similarly to the HMMM model,^17^ the head and tail groups in our split model can be reunited to make the standard lipid model after necessary equilibration or sampling. Another difference between the HMMM model and our split model is that of the primary model. HMMM is based on the CHARMM36 FF, while we used the Slipids FF. However, the same method for splitting the lipids could also be applied to the CHARMM36 FF and other FFs. In addition, our split model keeps the lipid tails intact, providing a more realistic environment compared with the HMMM model. The split model thus could be suitable for the study of transmembrane proteins, which is supported by our study of the EGFRtm dimer and GPCR. Moreover, the split model is designed so that researchers can easily expand it to include any type of lipid, going beyond the current five lipid types. We have implemented this model using only a handful of straightforward scripts, which we freely provide on *GitHub*.

An alternative approach to enhance lipid sampling is the MDAS method developed by Elber and Tieleman.^15,16^ MDAS enhances sampling by lipid swap, followed by short equilibration. The advantage is that in the swap, one can exchange two lipids, which are very far away and would require a very long time for diffusion. However, due to a low acceptance probability, the MDAS algorithm for all-atom FFs is currently computationally demanding,^16^ whereas our split model is efficient and can be employed for protein–lipid interactions and system equilibration at low computational cost. Promising results have been achieved with the current version of the split model. Moreover, the current version of the split model supports PSM, POPC, POPS, POPE, POPA, and cholesterol, but it can be straightforwardly extended to other types of lipids.

Note that faster diffusion of lipids could also be obtained by heating the membrane to a higher temperature. However, increased temperature also leads to an increase in the area per lipid and changes in the density profiles.

## Conclusions

We introduced a new “split” all-atom model to accelerate lipid diffusion in the molecular dynamics simulation of phospholipids membranes. The split model divides the phospholipid molecules into two moieties: the head and tail. To substitute the chemical bond holding the headgroups and tails together, external laterally uniform potentials were used to keep the headgroups in the position corresponding to nonsplit planar lipid membranes. Similarly, an external potential was applied to oxygen atoms of water to keep them in the bulk of the solvent. To prevent the acyl tails from flip-flopping, we also used an external potential on the first carbon atom of the tails. Our current split model supports membranes containing PSM, POPC, POPS, POPE, POPA, and CHL, although it can be readily developed for other lipid types.

We validated the interaction of the split model membrane with the Disheveled DEP domain and the amphiphilic helix in Opi1 as representatives for peripheral proteins and the EGFRtm dimer and the Human A2A Adenosine of G protein-coupled receptors (GPCRs) as representatives for transmembrane proteins. In comparison to the standard Slipids FF, the split Slipids force field (FF) produces ensembles with a higher number of protein–lipid headgroup interactions. The interaction decreased once the lipids were reunited. Moreover, our split FF correctly predicted the interaction sites of the proteins and the preferred lipid type. The split model produced a protein structure with a small deviation of the crystal structure. It is important to note that our primary focus was on the efficacy of the split model in differentiating between various lipids in relation to protein interactions rather than on the nuances of these interactions.

The split model enhances lateral lipid diffusion roughly 10 times. Therefore, one can equilibrate a large system comprising different phospholipid types at affordable computational costs. After equilibration, the lipids could be reunited into the standard membrane. It is important to note that after the reuniting process, system minimization and equilibration are necessary. Our open-source Split-Maker script, the Python code to reassemble the split lipids, and all necessary files to generate the split membranes are available online on *GitHub*.

## Data Availability

Input files for all simulations are available in the Zenodo repository at 10.5281/zenodo.13769594. All scripts and all necessary files are available online at https://github.com/Mehrnoosh64/Split_Membrane.git.

## Supporting Information Available

POPC and POPC/POPS area per lipid, average P–N angle histograms, and thickness (Figure S1); POPC and POPC/POPS membrane density profiles for different flat-bottomed restraining parameters (Figure S2); analysis of short-range interactions of POPC and DEP (Figure S3); analysis of short-range interactions of POPC/POPS and DEP (Figure S4); fluctuations of the helices3 COM of the DEP in POPC and POPC/POPS (Figure S5); RDF analysis of the DEP (Figure S6); the orientations of DEP domains on POPC (Figure S7); RDF analysis of Opi1 (Figure S8); RMSD of the A2A adenosine receptor (Figure S9); RDF analysis of GPCR (Figure S10); density profiles for different lipids of plasma membrane mimics (Figure S11); RDF for different lipids of plasma membrane mimics (Figure S12); and density profile and RDF for plasma membrane containing cholesterol (Figure S13) (PDF)

## References

[ref1] Pogozheva I. D.; Armstrong G. A.; Kong L.; Hartnagel T. J.; Carpino C. A.; Gee S. E.; Picarello D. M.; Rubin A. S.; Lee J.; Park S.; Lomize A. L.; Im W. Comparative Molecular Dynamics Simulation Studies of Realistic Eukaryotic, Prokaryotic, and Archaeal Membranes. J. Chem. Inf. Model. 2022, 62, 1036–1051. 10.1021/acs.jcim.1c01514. 35167752

[ref2] Ingólfsson H. I.; Carpenter T. S.; Bhatia H.; Bremer P.-T.; Marrink S. J.; Lightstone F. C. Computational lipidomics of the neuronal plasma membrane. Biophys. J. 2017, 113, 2271–2280. 10.1016/j.bpj.2017.10.017. 29113676 PMC5700369

[ref3] Bretscher M. S. Asymmetrical Lipid Bilayer Structure for Biological Membranes. Nat. New Biol. 1972, 236, 11–12. 10.1038/newbio236011a0. 4502419

[ref4] Zachowski A. Phospholipids in animal eukaryotic membranes: transverse asymmetry and movement. Biochem. J. 1993, 294, 1–14. 10.1042/bj2940001. 8363559 PMC1134557

[ref5] Balla T. Phosphoinositides: Tiny Lipids With Giant Impact on Cell Regulation. Physiol. Rev. 2013, 93, 1019–1137. 10.1152/physrev.00028.2012. 23899561 PMC3962547

[ref6] Wang X.; Devaiah S.; Zhang W.; Welti R. Signaling functions of phosphatidic acid. Prog. Lipid Res. 2006, 45, 250–278. 10.1016/j.plipres.2006.01.005. 16574237

[ref7] Marrink S. J.; Corradi V.; Souza P. C.; Ingólfsson H. I.; Tieleman D. P.; Sansom M. S. Computational Modeling of Realistic Cell Membranes. Chem. Rev. 2019, 119, 6184–6226. 10.1021/acs.chemrev.8b00460. 30623647 PMC6509646

[ref8] Ingólfsson H. I.; Melo M. N.; van Eerden F. J.; Arnarez C.; Lopez C. A.; Wassenaar T. A.; Periole X.; de Vries A. H.; Tieleman D. P.; Marrink S. J. Lipid Organization of the Plasma Membrane. J. Am. Chem. Soc. 2014, 136, 14554–14559. 10.1021/ja507832e. 25229711

[ref9] Singharoy A.; Maffeo C.; Delgado-Magnero K. H.; et al. Atoms to Phenotypes: Molecular Design Principles of Cellular Energy Metabolism. Cell 2019, 179 (5), 1098–1111. 10.2139/ssrn.3365009. 31730852 PMC7075482

[ref10] Bernardino de la Serna J.; Schütz G. J.; Eggeling C.; Cebecauer M. There Is No Simple Model of the Plasma Membrane Organization.. Front. Cell Dev. Biol. 2016, 4, 106 10.3389/fcell.2016.00106/full. 27747212 PMC5040727

[ref11] Lindblom G.; Orädd G. Lipid lateral diffusion and membrane heterogeneity. Biochim. Biophys. Acta, Biomembr. 2009, 1788, 234–244. 10.1016/j.bbamem.2008.08.016. 18805393

[ref12] Zhang J.; Li W.; Wang J.; Qin M.; Wu L.; Yan Z.; Xu W.; Zuo G.; Wang W. Protein folding simulations: From coarse-grained model to all-atom model. IUBMB Life 2009, 61, 627–643. 10.1002/iub.223. 19472192

[ref13] Jämbeck J. P. M.; Lyubartsev A. P. Derivation and Systematic Validation of a Refined All-Atom Force Field for Phosphatidylcholine Lipids. J. Phys. Chem. B 2012, 116, 3164–3179. 10.1021/jp212503e. 22352995 PMC3320744

[ref14] Marrink S. J.; Tieleman D. P. Perspective on the Martini model. Chem. Soc. Rev. 2013, 42, 6801–6822. 10.1039/c3cs60093a. 23708257

[ref15] Fathizadeh A.; Elber R. A mixed alchemical and equilibrium dynamics to simulate heterogeneous dense fluids: Illustrations for Lennard-Jones mixtures and phospholipid membranes. J. Chem. Phys. 2018, 149, 072325 10.1063/1.5027078. 30134684 PMC6018062

[ref16] Cherniavskyi Y. K.; Fathizadeh A.; Elber R.; Tieleman D. P. Computer simulations of a heterogeneous membrane with enhanced sampling techniques. J. Chem. Phys. 2020, 153, 144110 10.1063/5.0014176. 33086798 PMC7556882

[ref17] Tajkhorshid E. Accelerating Membrane Insertion of Peripheral Proteins with a Novel Membrane Mimetic Model. Biophys. J. 2012, 102, 6a 10.1016/j.bpj.2011.11.054. PMC3341550 22824277

[ref18] Vermaas J. V.; Pogorelov T. V.; Tajkhorshid E. Extension of the Highly Mobile Membrane Mimetic to Transmembrane Systems Through Customized in silico Solvents. J. Phys. Chem. B 2017, 121, 3764–3776. 10.1021/acs.jpcb.6b11378. 28241729 PMC5558153

[ref19] Baylon J. L.; Lenov I. L.; Sligar S. G.; Tajkhorshid E. Characterizing the Membrane-Bound State of Cytochrome P450 3A4: Structure, Depth of Insertion, and Orientation. J. Am. Chem. Soc. 2013, 135, 8542–8551. 10.1021/ja4003525. 23697766 PMC3682445

[ref20] Pogorelov T. V.; Vermaas J. V.; Arcario M. J.; Tajkhorshid E. Partitioning of Amino Acids into a Model Membrane: Capturing the Interface. J. Phys. Chem. B 2014, 118, 1481–1492. 10.1021/jp4089113. 24451004 PMC3983343

[ref21] Qi Y.; Cheng X.; Lee J.; Vermaas J. V.; Pogorelov T. V.; Tajkhorshid E.; Park S.; Klauda J. B.; Im W. CHARMM-GUI HMMM Builder for Membrane Simulations with the Highly Mobile Membrane-Mimetic Model. Biophys. J. 2015, 109, 2012–2022. 10.1016/j.bpj.2015.10.008. 26588561 PMC4656882

[ref22] Abraham M. J.; Murtola T.; Schulz R.; Páll S.; Smith J. C.; Hess B.; Lindahl E. GROMACS: High performance molecular simulations through multi-level parallelism from laptops to supercomputers. SoftwareX 2015, 1–2, 19–25. 10.1016/j.softx.2015.06.001.

[ref23] Lindahl E.; Abraham M. J.; Hess B.; van der Spoel D. GROMACS Source code2021. 2021 10.5281/zenodo.4457626.

[ref24] Páll S.; Abraham M. J.; Kutzner C.; Hess B.; Lindahl E. Tackling Exascale Software Challenges in Molecular Dynamics Simulations with GROMACS. In Solving Software Challenges for Exascale: International Conference on Exascale Applications and Software, EASC 2014, Stockholm, Sweden, April 2-3, 2014, Revised Selected Papers 2; Springer International Publishing, 2015; pp 3–27 10.1007/978-3-319-15976-8_1.

[ref25] Bussi G.; Donadio D.; Parrinello M. Canonical sampling through velocity rescaling. J. Chem. Phys. 2007, 126, 014101 10.1063/1.2408420. 17212484

[ref26] Parrinello M.; Rahman A. Crystal Structure and Pair Potentials: A Molecular-Dynamics Study. Phys. Rev. Lett. 1980, 45, 1196–1199. 10.1103/PhysRevLett.45.1196.

[ref27] Darden T.; York D.; Pedersen L. Particle mesh Ewald: An N.log(N) method for Ewald sums in large systems. J. Chem. Phys. 1993, 98, 10089–10092. 10.1063/1.464397.

[ref28] Miyamoto S.; Kollman P. A. Settle: An analytical version of the SHAKE and RATTLE algorithm for rigid water models. J. Comput. Chem. 1992, 13, 952–962. 10.1002/jcc.540130805.

[ref29] Hess B.; Bekker H.; Berendsen H. J. C.; Fraaije J. G. E. M. LINCS: A linear constraint solver for molecular simulations. J. Comput. Chem. 1997, 18, 1463–1472. 10.1002/(SICI)1096-987X(199709)18:12<1463::AID-JCC4>3.0.CO;2-H.

[ref30] Lorent J. H.; Levental K. R.; Ganesan L.; Rivera-Longsworth G.; Sezgin E.; Doktorova M.; Lyman E.; Levental I. Plasma membranes are asymmetric in lipid unsaturation, packing and protein shape. Nat. Chem. Biol. 2020, 16, 644–652. 10.1038/s41589-020-0529-6. 32367017 PMC7246138

[ref31] Jo S.; Kim T.; Iyer V. G.; Im W. CHARMM-GUI: A web-based graphical user interface for CHARMM. J. Comput. Chem. 2008, 29, 1859–1865. 10.1002/jcc.20945. 18351591

[ref32] Brooks B. R.; Brooks C. L.; Mackerell A. D.; et al. CHARMM: The biomolecular simulation program. J. Comput. Chem. 2009, 30, 1545–1614. 10.1002/jcc.21287. 19444816 PMC2810661

[ref33] Lee J.; Cheng X.; Jo S.; MacKerell A. D.; Klauda J. B.; Im W. CHARMM-GUI Input Generator for NAMD, Gromacs, Amber, Openmm, and CHARMM/OpenMM Simulations using the CHARMM36 Additive Force Field. Biophys. J. 2016, 110, 641a 10.1016/j.bpj.2015.11.3431. PMC4712441 26631602

[ref34] Wu E. L.; Cheng X.; Jo S.; Rui H.; Song K. C.; Dávila-Contreras E. M.; Qi Y.; Lee J.; Monje-Galvan V.; Venable R. M.; Klauda J. B.; Im W. CHARMM-GUI Membrane Builder toward realistic biological membrane simulations. J. Comput. Chem. 2014, 35, 1997–2004. 10.1002/jcc.23702. 25130509 PMC4165794

[ref35] Klauda J. B.; Venable R. M.; Freites J. A.; O’Connor J. W.; Tobias D. J.; Mondragon-Ramirez C.; Vorobyov I.; MacKerell A. D.; Pastor R. W. Update of the CHARMM All-Atom Additive Force Field for Lipids: Validation on Six Lipid Types. J. Phys. Chem. B 2010, 114, 7830–7843. 10.1021/jp101759q. 20496934 PMC2922408

[ref36] Jämbeck J. P. M.; Lyubartsev A. P. An Extension and Further Validation of an All-Atomistic Force Field for Biological Membranes. J. Chem. Theory Comput. 2012, 8, 2938–2948. 10.1021/ct300342n. 26592132

[ref37] Jämbeck J. P. M.; Lyubartsev A. P. Exploring the Free Energy Landscape of Solutes Embedded in Lipid Bilayers. J. Phys. Chem. Lett. 2013, 4, 1781–1787. 10.1021/jz4007993. 26283109

[ref38] Rose M.; Hirmiz N.; Moran-Mirabal J.; Fradin C. Lipid Diffusion in Supported Lipid Bilayers: A Comparison between Line-Scanning Fluorescence Correlation Spectroscopy and Single-Particle Tracking. Membranes 2015, 5, 702–721. 10.3390/membranes5040702. 26610279 PMC4704007

[ref39] Falginella F. L.; Kravec M.; Drabinová M.; Paclíková P.; Bryja V.; Vácha R. Binding of DEP domain to phospholipid membranes: More than just electrostatics. Biochim. Biophys. Acta, Biomembr. 2022, 1864, 183983 10.1016/j.bbamem.2022.183983. 35750206

[ref40] Yu A.; Xing Y.; Harrison S. C.; Kirchhausen T. Structural Analysis of the Interaction between Dishevelled2 and Clathrin AP-2 Adaptor, A Critical Step in Noncanonical Wnt Signaling. Structure 2010, 18, 1311–1320. 10.1016/j.str.2010.07.010. 20947020 PMC2992793

[ref41] Schrödinger L.; DeLano W. Pymol 2020 http://www.pymol.org/pymol.

[ref42] Case D. AMBER 2018; University of California: San Francisco, 2018.

[ref43] Lindorff-Larsen K.; Piana S.; Palmo K.; Maragakis P.; Klepeis J. L.; Dror R. O.; Shaw D. E. Improved side-chain torsion potentials for the Amber ff99SB protein force field. Proteins 2010, 78, 1950–1958. 10.1002/prot.22711. 20408171 PMC2970904

[ref44] Rosenbaum D. M.; Rasmussen S.; Kobilka B. The structure and function of G-protein-coupled receptors. Nature 2009, 459, 356–363. 10.1038/nature08144. 19458711 PMC3967846

[ref45] Wei S.; Thakur N.; Ray A.; Jin B.; Obeng S.; McCurdy C.; McMahon L.; de Terán H. G.; Eddy M.; Lamichhane R. Slow conformational dynamics of the human A2A adenosine receptor are temporally ordered. Structure 2022, 30, 329–337. 10.1016/j.str.2021.11.005. 34895472 PMC8897252

[ref46] Jaakola V.-P.; Griffith M.; Hanson M.; Cherezov V.; Chien E.; Lane J.; IJzerman A.; Stevens R. The 2.6 Angstrom Crystal Structure of a Human A2A Adenosine Receptor Bound to an Antagonist. Science 2008, 322, 1211–1217. 10.1126/science.1164772. 18832607 PMC2586971

[ref47] Li L.; Vorobyov I.; Allen T. The Different Interactions of Lysine and Arginine Side Chains with Lipid Membranes. J. Phys. Chem. B 2013, 117, 11906–11920. 10.1021/jp405418y. 24007457 PMC6548679

[ref48] Bocharov E. V.; Bragin P. E.; Pavlov K. V.; Bocharova O. V.; Mineev K. S.; Polyansky A. A.; Volynsky P. E.; Efremov R. G.; Arseniev A. S. The Conformation of the Epidermal Growth Factor Receptor Transmembrane Domain Dimer Dynamically Adapts to the Local Membrane Environment. Biochemistry 2017, 56, 1697–1705. 10.1021/acs.biochem.6b01085. 28291355

[ref49] Arkhipov A.; Shan Y.; Das R.; Endres N. F.; Eastwood M. P.; Wemmer D. E.; Kuriyan J.; Shaw D. E. Architecture and Membrane Interactions of the EGF Receptor. Cell 2013, 152, 557–569. 10.1016/j.cell.2012.12.030. 23374350 PMC3680629

[ref50] Gullingsrud J.; Schulten K. Lipid Bilayer Pressure Profiles and Mechanosensitive Channel Gating. Biophys. J. 2004, 86, 3496–3509. 10.1529/biophysj.103.034322. 15189849 PMC1304254

[ref51] Shahane G.; Ding W.; Palaiokostas M.; Orsi M. Physical properties of model biological lipid bilayers: insights from all-atom molecular dynamics simulations. J. Mol. Model. 2019, 25, 76 10.1007/s00894-019-3964-0. 30806797

[ref52] Yoo J.; Aksimentiev A. Improved Parameterization of Amine–Carboxylate and Amine–Phosphate Interactions for Molecular Dynamics Simulations Using the CHARMM and AMBER Force Fields. J. Chem. Theory Comput. 2016, 12, 430–443. 10.1021/acs.jctc.5b00967. 26632962

[ref53] Yoo J.; Aksimentiev A. New tricks for old dogs: improving the accuracy of biomolecular force fields by pair-specific corrections to non-bonded interactions. Phys. Chem. Chem. Phys. 2018, 20, 8432–8449. 10.1039/C7CP08185E. 29547221 PMC5874203

