# Supplementary material for: Split Membrane: A New Model to Accelerate All-Atom MD Simulation of Phospholipid Bilayers
Source: J Chem Inf Model. 2025 Jan 8;65(2):845–56. doi: 10.1021/acs.jcim.4c01664 (PMC11776049; doi:10.1021/acs.jcim.4c01664)
Supplement: Supplementary file 1 — ci4c01664_si_001.pdf [file ci4c01664_si_001.pdf]

Supporting Information

Split Membrane - A New Model to Accelerate All-atom MD Simulation of Phospholipid Bilayers

Mehrnoosh Khodam Hazrati[†], Lukáš Sukeník[†,‡], and Robert Vácha[*,†,‡,¶]

[†] CEITEC – Central European Institute of Technology, Masaryk University, Kamenice 753/5, 625 00 Brno, Czech Republic
[‡] National Centre for Biomolecular Research, Faculty of Science, Masaryk University, Kamenice 5, 625 00 Brno, Czech Republic
[¶] Department of Condensed Matter Physics, Faculty of Science, Masaryk University, Kotlářská 267/2, 611 37 Brno, Czech Republic

E-mail: robert.vacha@muni.cz

Table S1: Average coulombic and Lennard-Jones short-range interaction energies between DEP domain disheveled protein and POPC headgroups in the pure POPC, pure split POPC, and pure reunited POPC systems.

	Coulomb(kJ/mol)	LJ(kJ/mol)
POPC : DEP	-4.12(±1.39)	-1.76(±0.50)
Split POPC : DEP	-12.89(±0.83)	-3.88(±0.03)
Reunited POPC : DEP	-7.73(±3.99)	-2.52(±0.79)

Table S2: Sum of coulombic and Lennard-Jones short-range interaction energies between DEP domain disheveled protein and POPC headgroups in the pure POPC, pure split POPC, and pure reunited POPC systems.

	Coulomb(kJ/mol)	LJ(kJ/mol)
POPC : DEP	-358.04(±120.69)	-153.26(±43.15)
Split POPC : DEP	-1121.76(±71.89)	-337.96(±2.52)
Reunited POPC :DEP	-672.81(±347.26)	-219.61(±69.19)

Table S3: Average coulombic and Lennard-Jones short-range interaction energies between DEP domain disheveled protein and POPC/POPS headgroups in the binary POPC/POPS, split POPC/POPS, and reunited POPC/POPS systems.

	Coulomb(kJ/mol)	LJ(kJ/mol)
POPC : DEP	-2.88(±1.76)	-1.26(±0.56)
Split POPC : DEP	-4.91(±0.90)	-1.85(±0.40)
Reunited POPC : DEP	-4.04(±0.05)	-1.49(±0.09)
POPS : DEP	-10.81(±2.64)	-1.51(±0.48)
Split POPS : DEP	-11.50(±1.47)	-1.75(±0.16)
Reunited POPS : DEP	-11.64(±2.39)	-1.65(±0.42)

Table S4: Sum of coulombic and Lennard-Jones short-range interaction energies between DEP domain disheveled protein and POPC/POPS headgroups in the binary POPC/POPS and split POPC/POPS systems.

	Coulomb(kJ/mol)	LJ(kJ/mol)
POPC : DEP	-250.32(±153.01)	-109.90(±49.10)
Split POPC : DEP	-427.39(±78.26)	-160.63(±34.90)
Reunited POPC : DEP	-351.26(±4.36)	-129.29(±7.50)
POPS : DEP	-940.61(±229.36)	-131.88(±42.14)
Split POPS : DEP	-1000.37(±128.30)	-152.09(±14.02)
Reunited POPS : DEP	-1012.74(±207.75)	-143.45(±36.43)

Table S5: Diffusion coefficients of the headgroups in the outer and scrambled mimics for the split PMs compared to the corresponding PMs (nm^2/ns).

D×10^2(nm^2/ns)				
System	Lipid Type	Standard Slipids FF	Split Slipids FF	Speed-up
outer	SM	0.31(±0.05)	8.37(±0.07)	27
	PC	0.33(±0.02)	8.12(±0.12)	25
	PS	0.26(±0.14)	11.56(±0.85)	44
	PA	0.20(±0.15)	13.25(±0.16)	66
scr.	SM	0.36(±0.08)	8.26(±0.12)	23
	PC	0.42(±0.06)	8.86(±0.57)	21
	PS	0.37(±0.03)	11.64(±0.29)	31
	PE	0.36(±0.08)	3.68(±0.02)	10
	PA	0.22(±0.04)	11.21(±0.89)	51

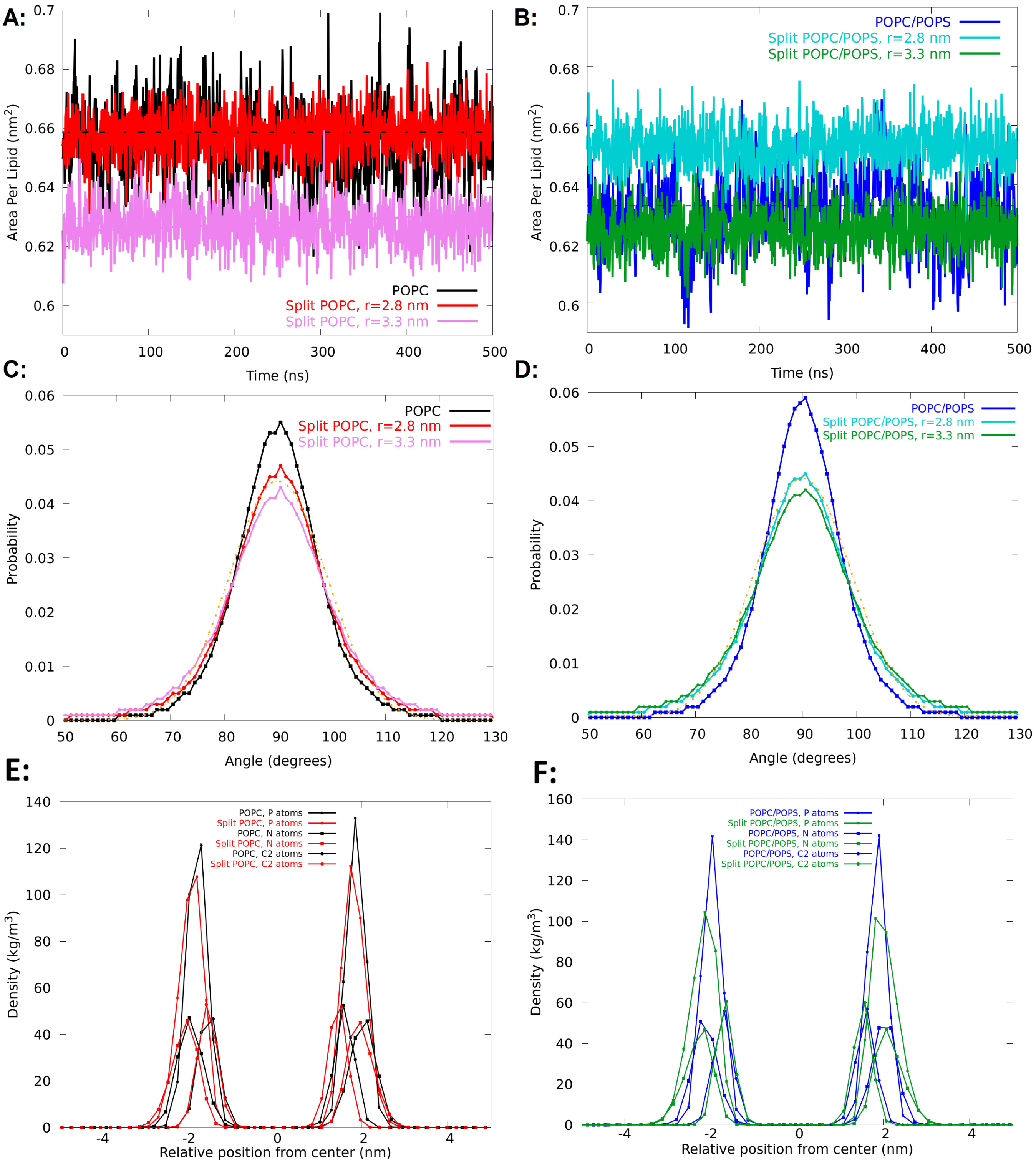

Figure S1: Area per lipid during 500 ns of the simulation time for A: POPC and B:POPC/POPS. C and D: Average P-N angle histograms with respect to the normal of pure POPC and binary POPC/POPS, respectively. Orange dash lines indicate the Gaussian distribution. E and F: Comparison of membrane thickness using P and N atoms of the headgroups, and C2 atoms of the tails as references for the standard and split models of pure POPC and binary POPC/POPS, respectively.

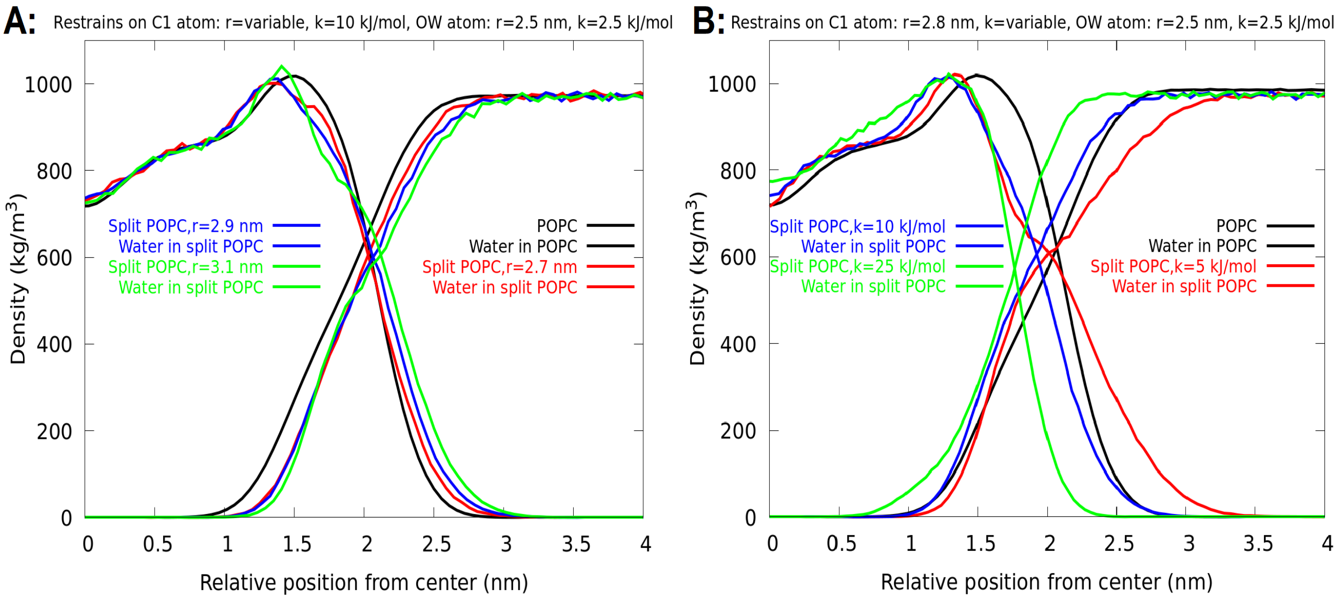

Figure S2: Comparison of membrane density profiles, illustrating the effects of A: varied flat-bottomed restraining radii (r) and B: different force constants (k) on membrane structure.

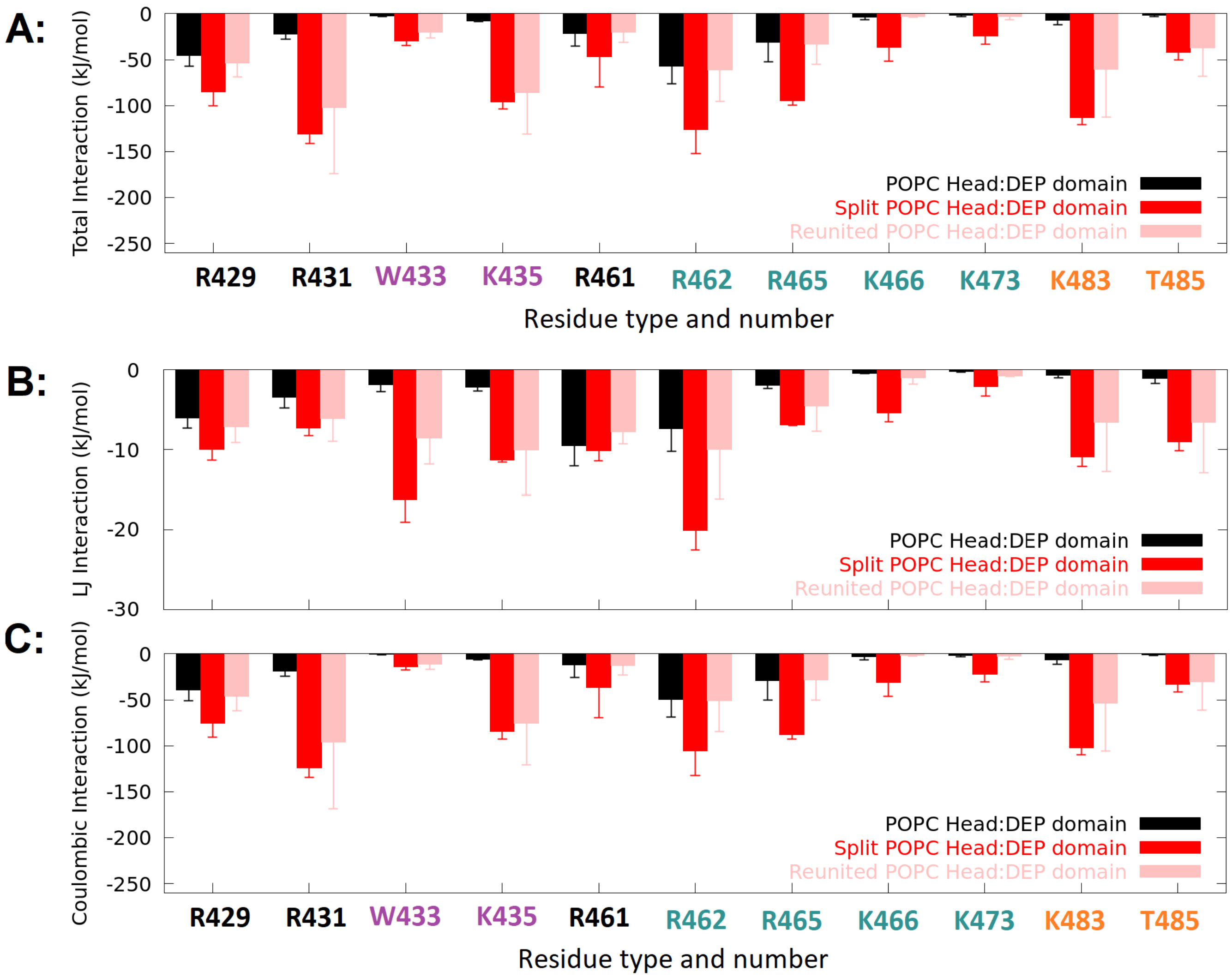

Figure S3: A: Analysis of average total (LJ + Coulombic) short-range interactions of the most interacting residues of DEP domain with POPC headgroups in the pure POPC systems. B: Lennard-Jones and C: Coulombic interaction potentials of the most interacting residues of DEP domain with POPC headgroups in the pure POPC systems.

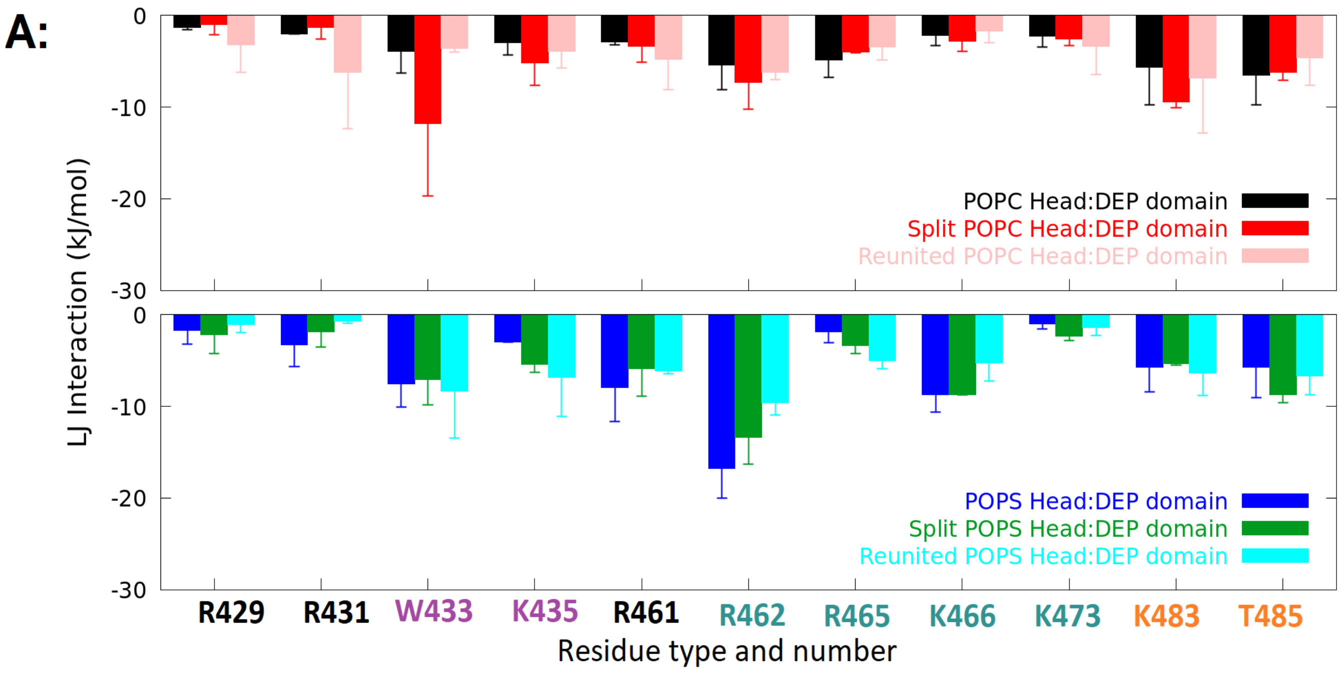

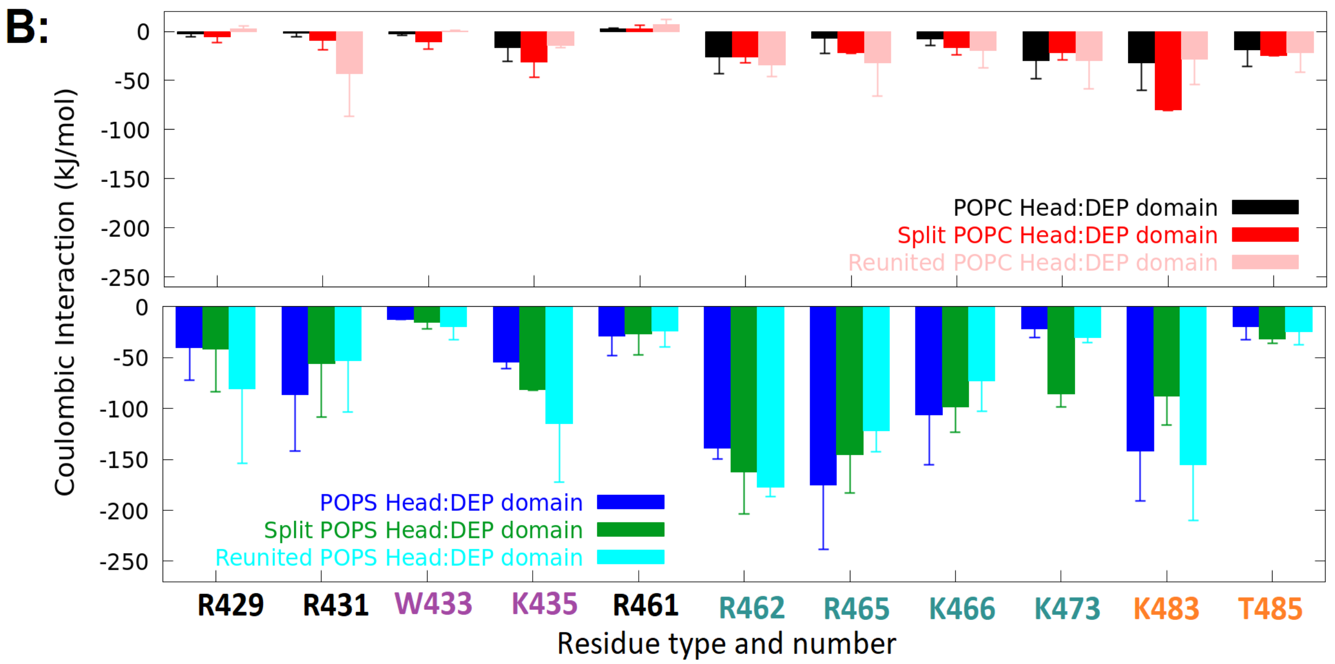

Figure S4: A: Lennard-Jones and B: Coulombic short-range interaction potentials of the most interacting residues of DEP domain with POPC headgroups as well as POPS headgroups in the binary POPC/POPS systems.

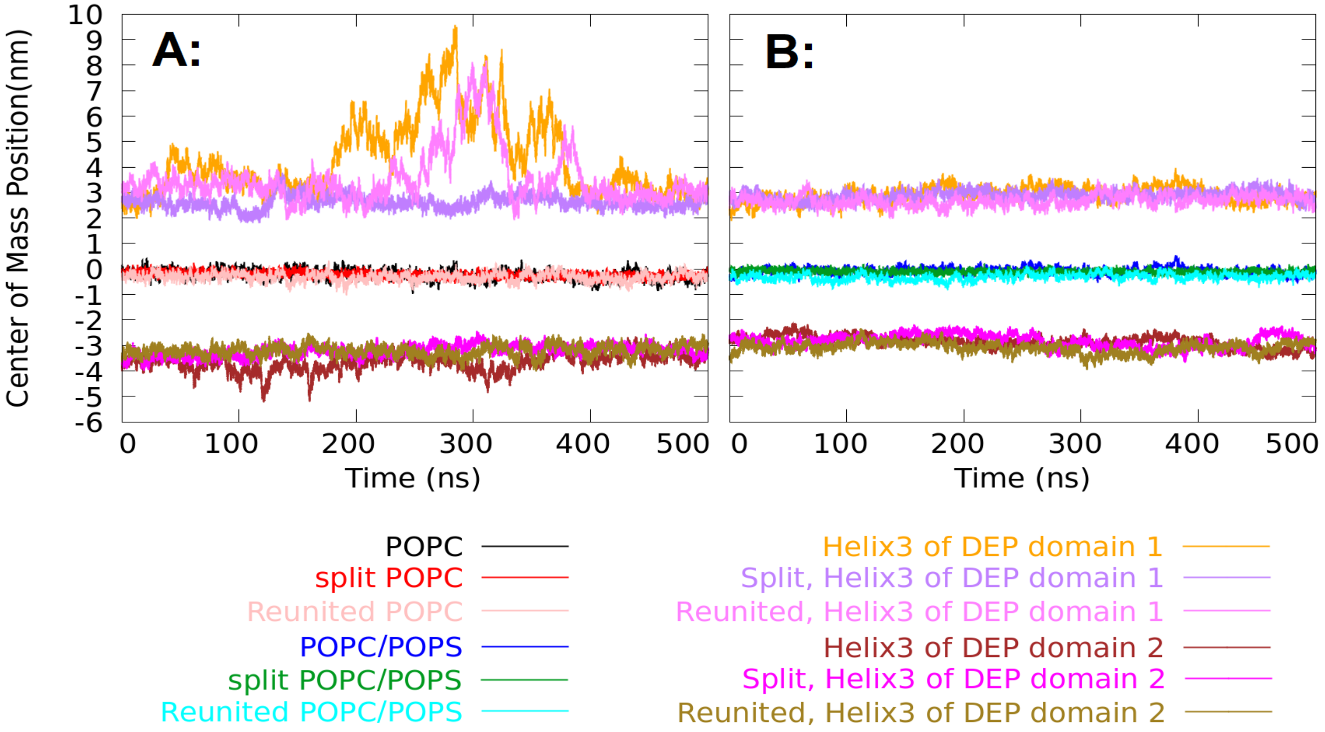

Figure S5: Fluctuations of the helices3 COM of the DEP domains and the membranes in A: pure POPC and B: binary POPC/POPS systems over the 500 ns of the simulation time.

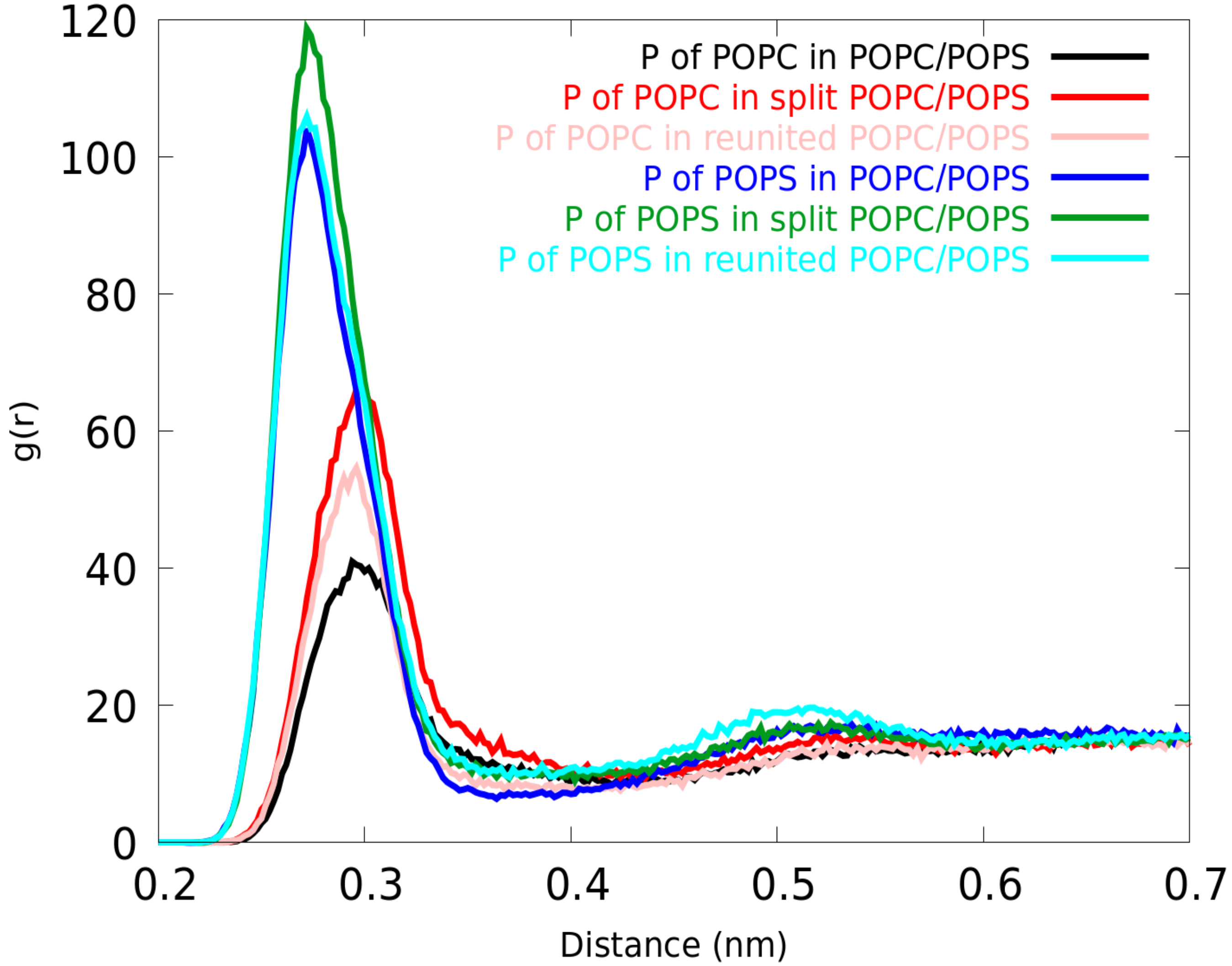

Figure S6: RDF analysis between the DEP domain surface and P atoms of POPC and POPS in POPC/POPS, Split POPC/POPS, and reunited POPC/POPS.

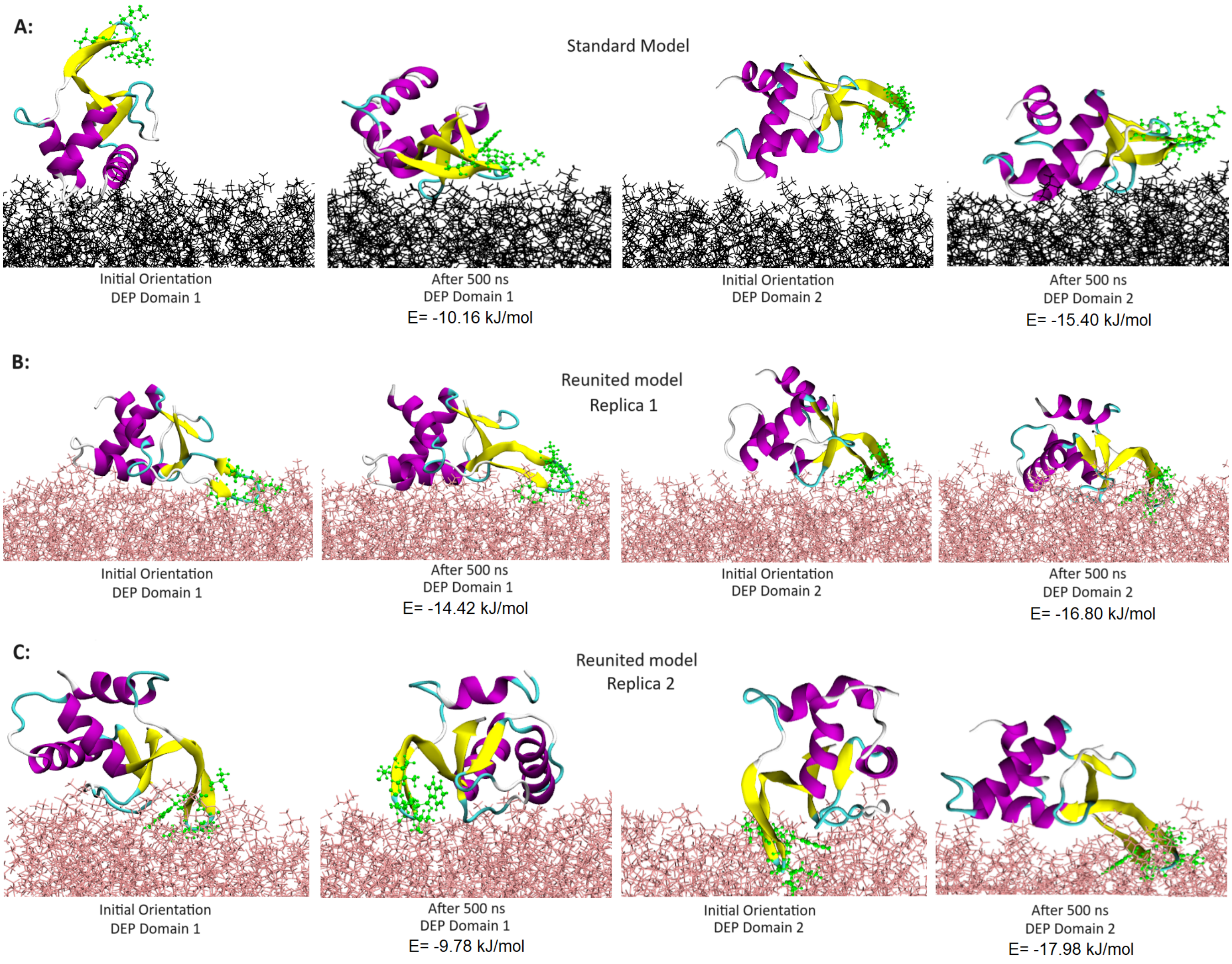

Figure S7: The orientations of DEP domains on POPC membranes including the initial configurations and configurations after 500 ns of simulation. A: the standard POPC, B: the reunited POPC replica 1, and C: the reunited POPC replica 2. There are two independent domains in each replica. Below figures we display the total short-range Coulomb and Lennard-Jones interaction energy, E, averaged over the last 50 ns. The finger loop of the DEP domains is shown in green CPK drawing mode to highlight the orientation of the domains. While DEP domain 2 in the standard POPC remained in the proximity of the membrane surface the whole time, DEP domain 1 in the standard and the reunited POPC replica 1 were completely desorbed and adsorbed again during the simulation time. We did not observe any desorption for other domains.

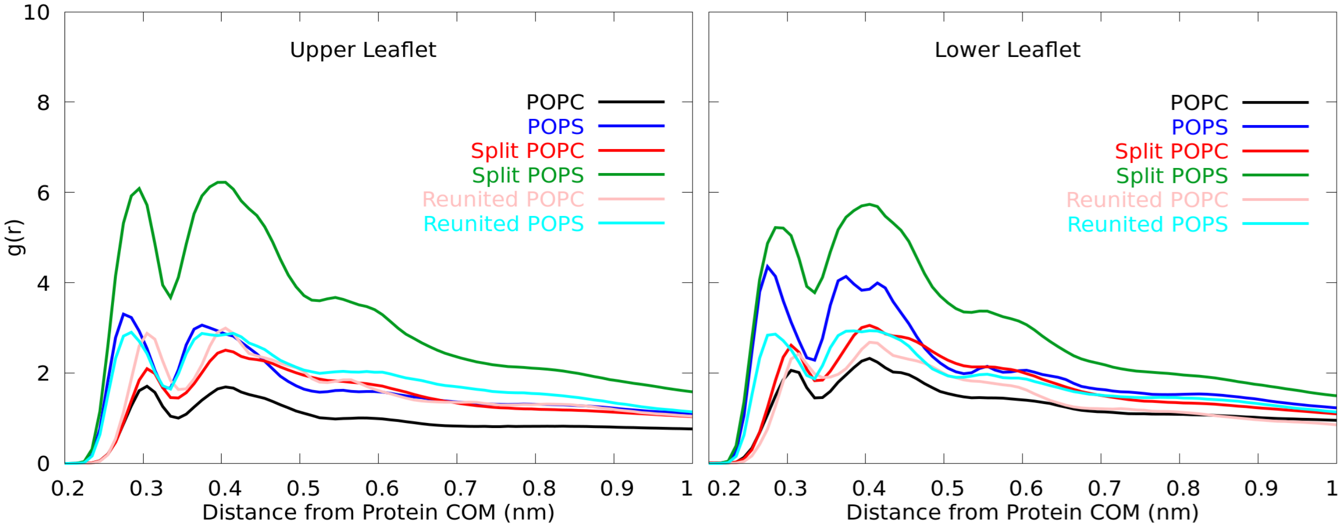

Figure S8: RDF analysis between the amphiphilic helix in Opi1 surface and P atoms of POPC and POPS in POPC/POPS, Split POPC/POPS, and reunited POPC/POPS.

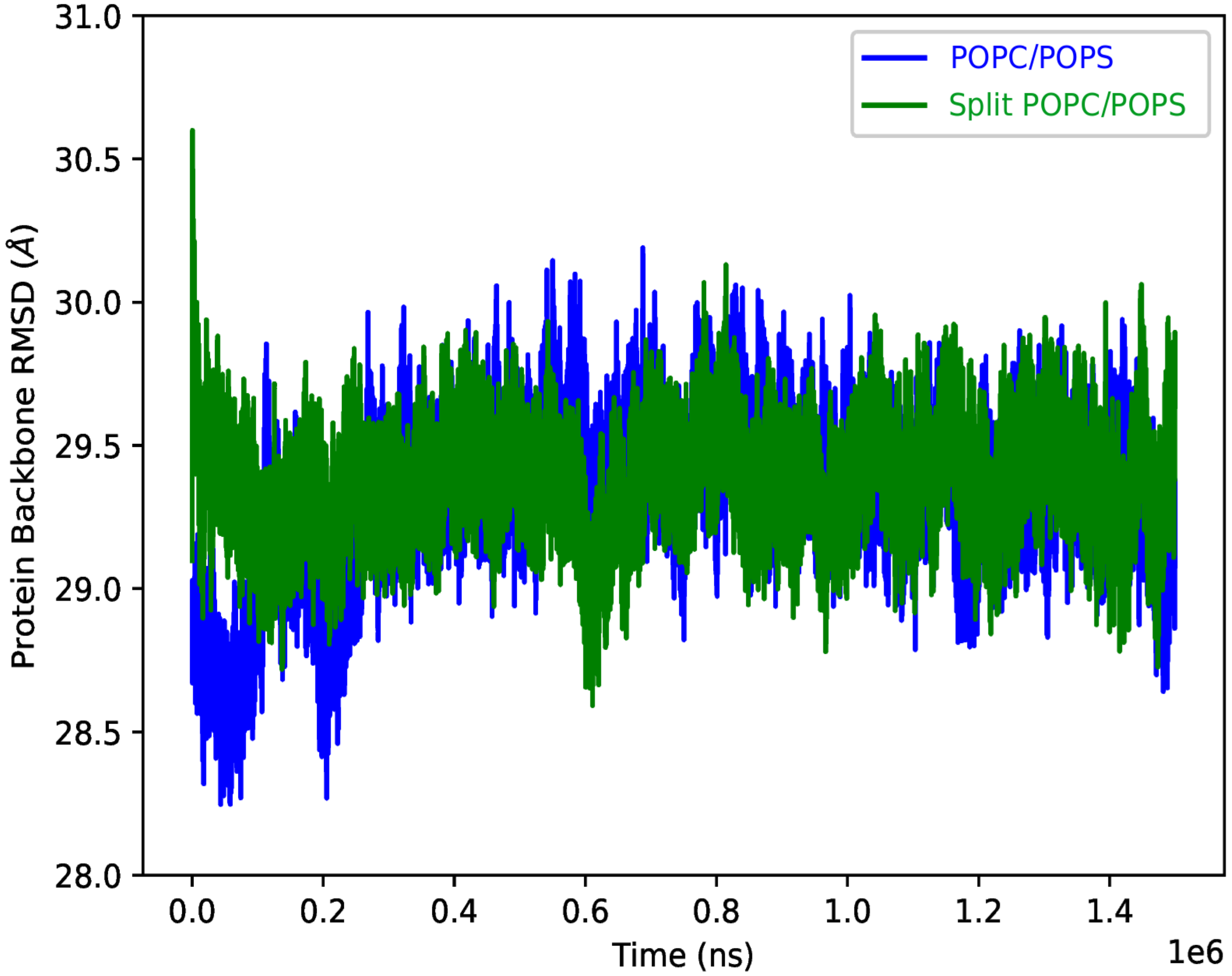

Figure S9: The backbone root-mean-square deviation (RMSD) of the A2A adenosine receptor in the standard and split POPC/POPS membranes.

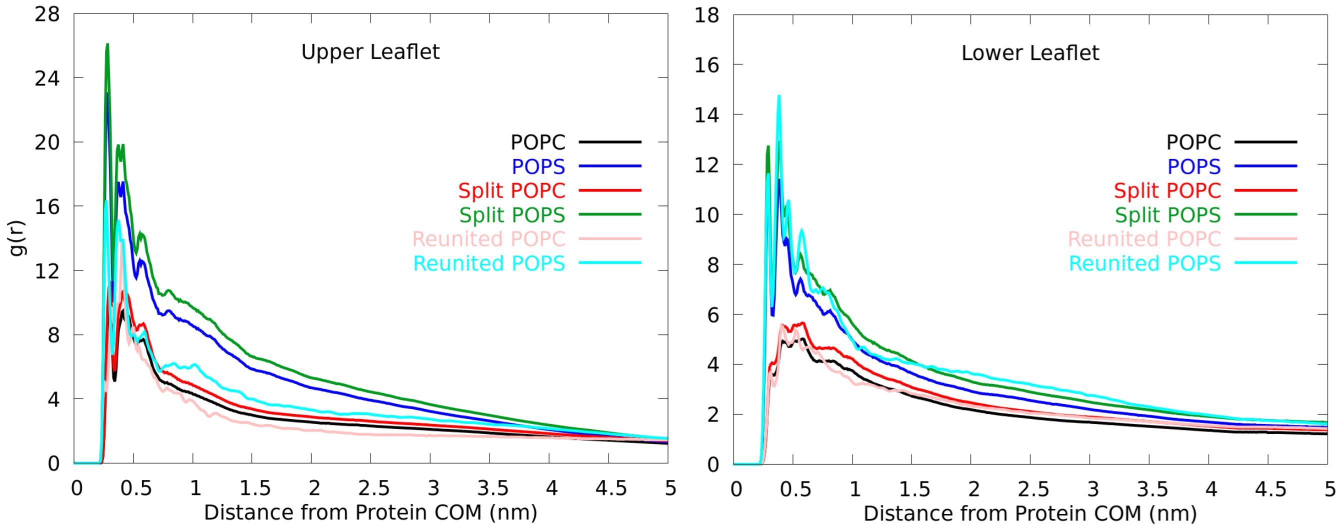

Figure S10: RDF analysis between the GPCR COM and P atoms of POPC and POPS in POPC/POPS, split POPC/POPS, and reunited POPC/POPS.

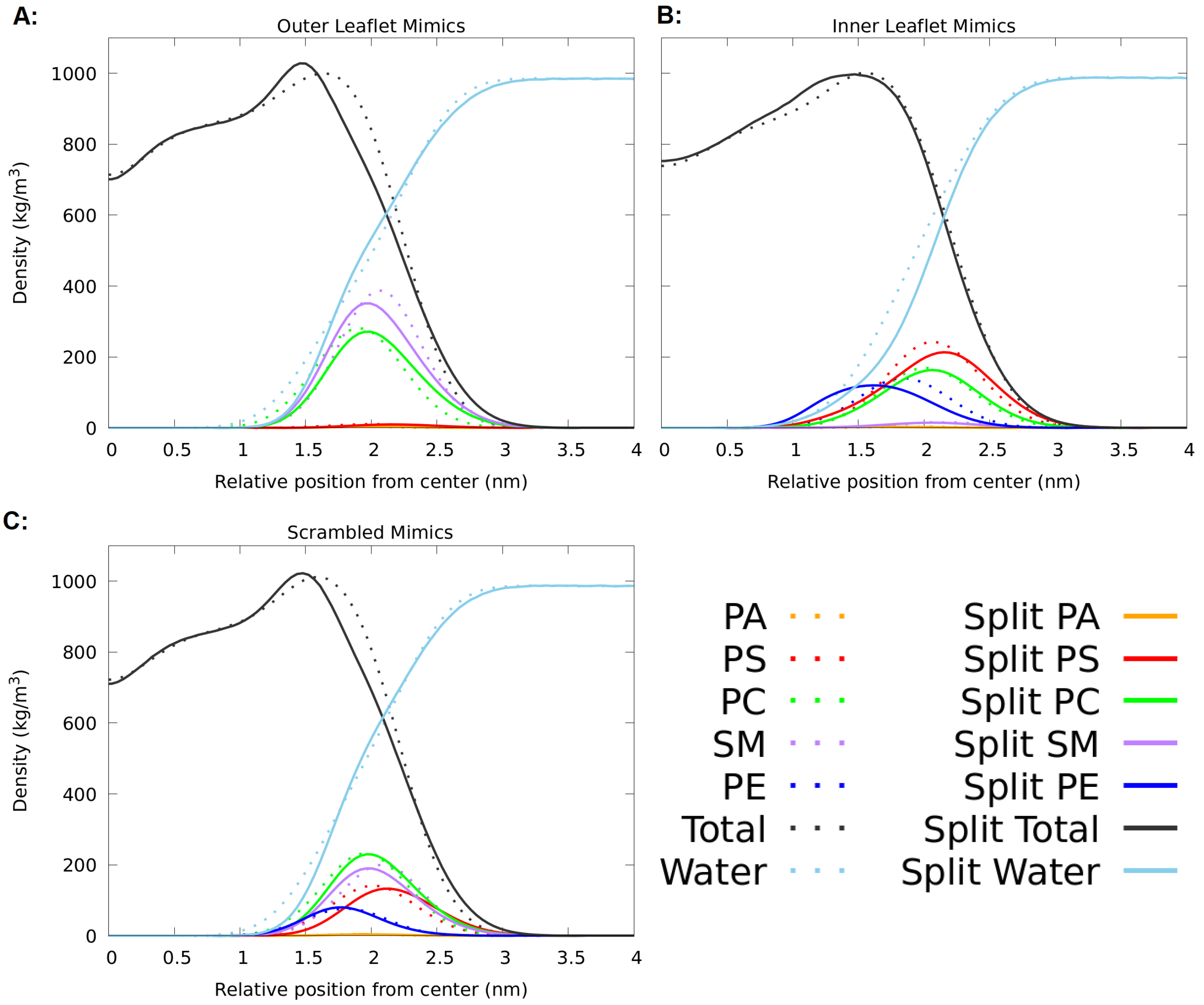

Figure S11: Density profiles for different groups in the PM mimics. A: outer leaflet, B: inner leaflet, and C: scrambled mimics.

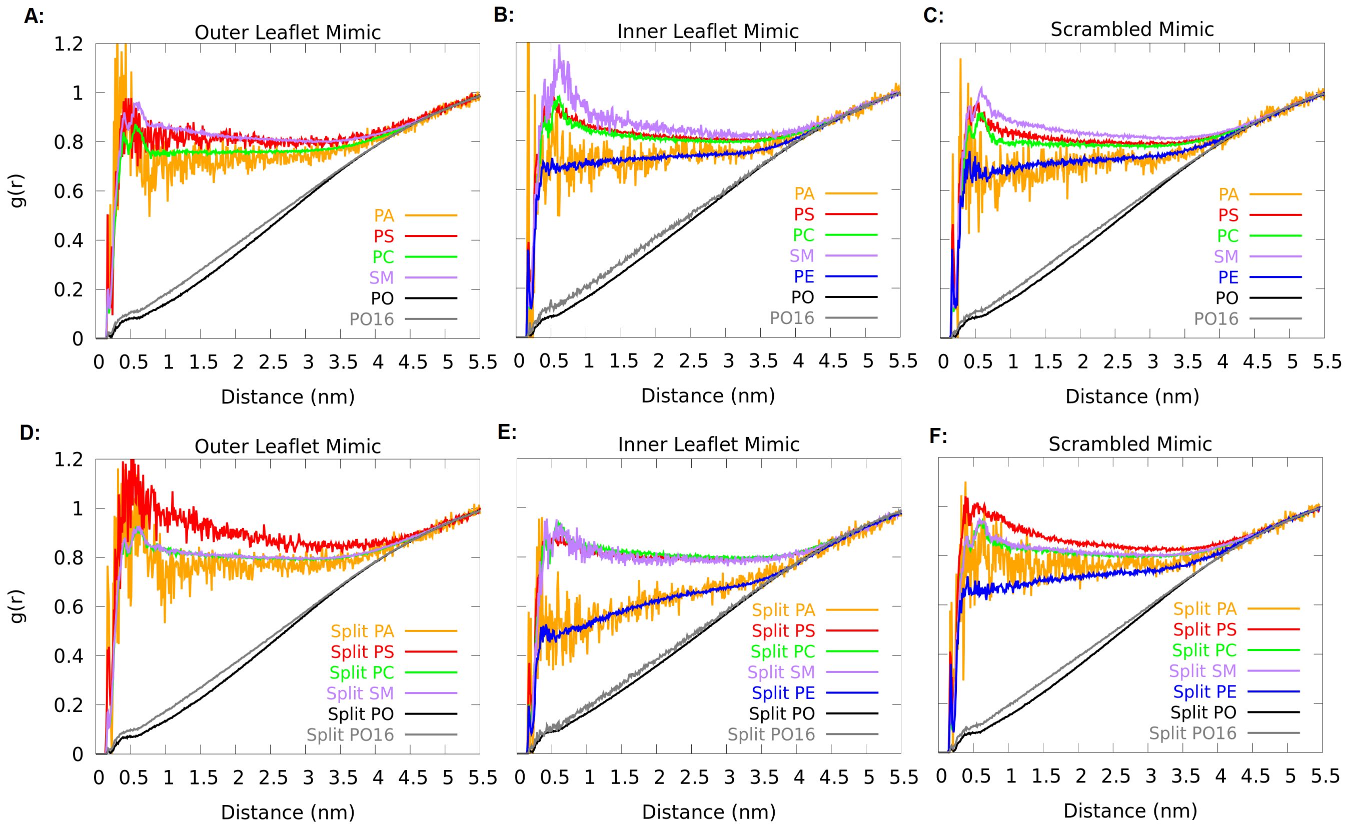

Figure S12: Radial distribution function (RDF) for different groups in the PM mimics with respect to water over the last frame simulated by A-C: Standard Slipids FF, D-F: Split Slipids FF. For better visualization, we depicted every 5 steps of the trajectories.

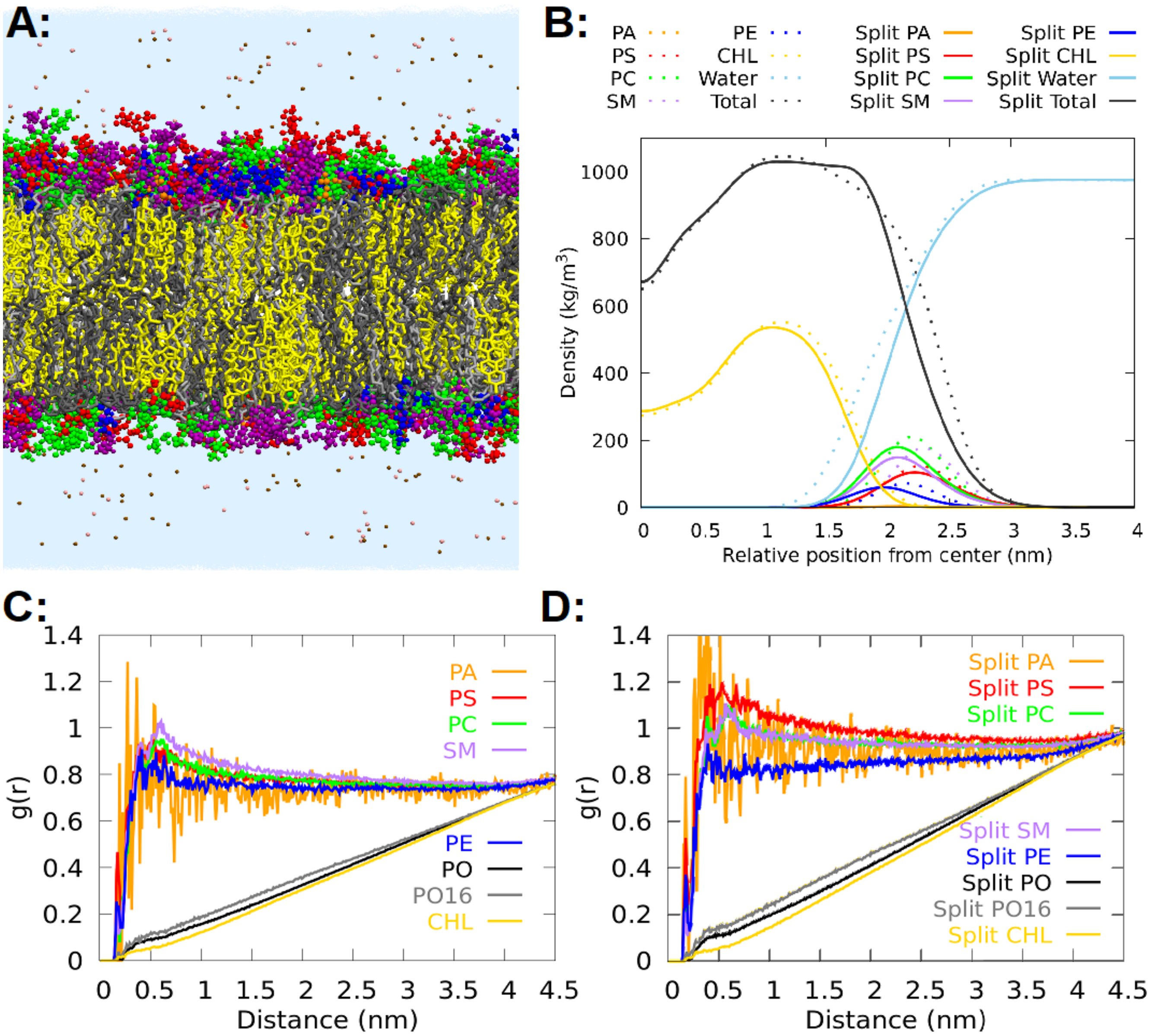

Figure S13: A: Side view snapshot of the split scrambled PM mimic containing 50% CHL after 1.5 μs of MD production run. Lipid colors are consistent with the RDF profiles. Ions are shown in pink and brown spheres, and water bulk in light blue. B: Density profiles for different groups with standard and split models. C, D: Radial distribution function (RDF) for different groups with respect to water over the last frame simulated by the standard and split models, respectively.
